# Water- and heat-activated dynamic passivation for perovskite photovoltaics

**DOI:** 10.1038/s41586-024-07705-5

**Published:** 2024-06-24

**Authors:** Wei-Ting Wang, Philippe Holzhey, Ning Zhou, Qiang Zhang, Suer Zhou, Elisabeth A. Duijnstee, Kevin J. Rietwyk, Jeng-Yu Lin, Yijie Mu, Yanfeng Zhang, Udo Bach, Chun-Guey Wu, Hin‐Lap Yip, Henry J. Snaith, Shien-Ping Feng

**Affiliations:** 1 Department of Systems Engineering, City University of Hong Kong, Kowloon, Hong Kong; 2 Clarendon Laboratory, Department of Physics, University of Oxford, Oxford, UK; 3 Australian Research Council Centre of Excellence in Exciton Science, Department of Chemical and Biological Engineering, Monash University, Clayton, Victoria Australia; 4 Department of Materials Science and Engineering, City University of Hong Kong, Kowloon, Hong Kong; 5 School of Chemistry, Xi’an Jiaotong University, Xi’an, China; 6 Department of Chemical and Materials Engineering, Tunghai University, Taichung City, Taiwan; 7 Department of Mechanical Engineering, The University of Hong Kong, Pok Fu Lam, Hong Kong; 8 Department of Chemistry and Research Center for New Generation Light Driven Photovoltaic Modules, National Central University, Taoyuan City, Taiwan; 9 School of Energy and Environment, City University of Hong Kong, Kowloon, Hong Kong; 10 Hong Kong Institute for Clean Energy, City University of Hong Kong, Kowloon, Hong Kong; 11 Center of Super-Diamond and Advanced Films, City University of Hong Kong, Kowloon, Hong Kong

**Keywords:** Electronic devices, Solar cells

## Abstract

Further improvements in perovskite solar cells require better control of ionic defects in the perovskite photoactive layer during the manufacturing stage and their usage^1–5^. Here we report a living passivation strategy using a hindered urea/thiocarbamate bond^6–8^ Lewis acid–base material (HUBLA), where dynamic covalent bonds with water and heat-activated characteristics can dynamically heal the perovskite to ensure device performance and stability. Upon exposure to moisture or heat, HUBLA generates new agents and further passivates defects in the perovskite. This passivation strategy achieved high-performance devices with a power conversion efficiency (PCE) of 25.1 per cent. HUBLA devices retained 94 per cent of their initial PCE for approximately 1,500 hours of ageing at 85 degrees Celsius in nitrogen and maintained 88 per cent of their initial PCE after 1,000 hours of ageing at 85 degrees Celsius and 30 per cent relative humidity in air.

## Abstract (web summary)

A living passivation strategy enables dynamic self-healing of perovskite solar cells to ensure device performance and stability on exposure to heat or moisture.

## Main

Perovskites are arguably the most promising next-generation photovoltaic materials as single-junction perovskite solar cells (PSCs) have reached a power conversion efficiency (PCE) of over 26% (ref. ^9^). The most critical challenge is improving the operational stability to reach lifetimes similar to silicon-based cells. Various passivation strategies (for example, lead oxysalt^10^, ionic liquids^11^, self-assembled monolayer^12^ and two-dimensional perovskite layer^13^) have been developed to improve the performance and reliability. However, environmental stress factors (for example, humidity, heat and light)^1–5^ cause the formation of traps or charge-carrier barriers within the perovskite absorber and deteriorate device performance. Most of the currently reported passivators interact with ionic defects and are then confined to certain locations after the manufacturing stage, making it difficult to passivate newly generated defects during device operation and storage. Ideally, the passivator not only enhances the endurance against environmental stressors but also can be activated by environmental stress factors to heal the perovskite dynamically. Here we propose a ‘living passivator’ containing dynamic covalent bonds (DCBs), which can be triggered by water and heat to release additional Lewis bases, thereby healing newly generated traps. Similar to the concept of living polymerization^14^, a protecting group (weak nucleophile) is used to form DCBs and quench the high reactivity of functional groups temporarily, as follows:

1 $${\rm{Electrophile}}+{\rm{Nucleophile}}\begin{array}{c}{k}_{1}\\ \rightleftarrows \\ {k}_{-1}\end{array}{\rm{Electrophile}}-{\rm{Nucleophile}}$$

where *k*_1_ represents the rate constant for the forward reaction and *k*_–1_ represents the rate constant for the reverse reaction. In contact with water, the highly reactive electrophiles accept electrons and release new passivators to interact with the perovskite. Moreover, heating accelerates the dynamic reaction of electrophile and nucleophile^15^.

## Design and dynamic reactions of HUBLA

Compared with most DCBs that rely on catalysts^16–21^, we chose the hindered urea bond (HUB) as a partial structure of the living passivator. In particular, the dynamic behaviour of HUB originates from the non-coplanarity of the amide bond (–N(R)C(O)–)^6,7^ (Supplementary Fig. 1), whose conjugation is disturbed by the introduction of the bulky *tert*-butyl structure (–C(CH_3_)_3_), enabling an intrinsic dissociation–association reaction at room temperature. To demonstrate this dynamic reaction of HUB, we fabricated a Rubik’s Cube-like structure out of 27 HUB-based elastomers (Supplementary Figs. 2 and 3, and Supplementary Note 1). The bond between the cubes was formed through the dissociation–association reaction of HUBs. In addition, we also integrated a thiocarbamate bond (–SC(O)N(H)–; denoted as TCB), which shows a dynamic behaviour at elevated temperatures^8^. The association–dissociation reaction of the two bonds can be expressed as follows.

The dynamic reaction of HUB at room temperature^6,7^:

2 $${{\rm{R}}}_{1}{\rm{NCO}}+\left({\left({{\rm{CH}}}_{3}\right)}_{3}{\rm{C}}\right){\rm{N}}({\rm{H}}){{\rm{R}}}_{2}\begin{array}{c}{k}_{1}\\ \rightleftarrows \\ {k}_{-1}\end{array}{{\rm{R}}}_{1}{\rm{N}}\left({\rm{H}}\right){\rm{C}}\left({\rm{O}}\right){\rm{N}}\left({\rm{C}}{\left({{\rm{CH}}}_{3}\right)}_{3}\right){{\rm{R}}}_{2}$$

The dynamic reaction of TCB at elevated temperature^8^:

3 $${{\rm{R}}}_{1}{\rm{NCO}}+{\rm{HS}}{{\rm{R}}}_{2}\begin{array}{c}{k}_{1}\\ \rightleftarrows \\ {k}_{-1}\end{array}{{\rm{R}}}_{1}{\rm{N}}({\rm{H}}){\rm{C}}({\rm{O}}){\rm{S}}{{\rm{R}}}_{2}$$

**Fig. 1:**
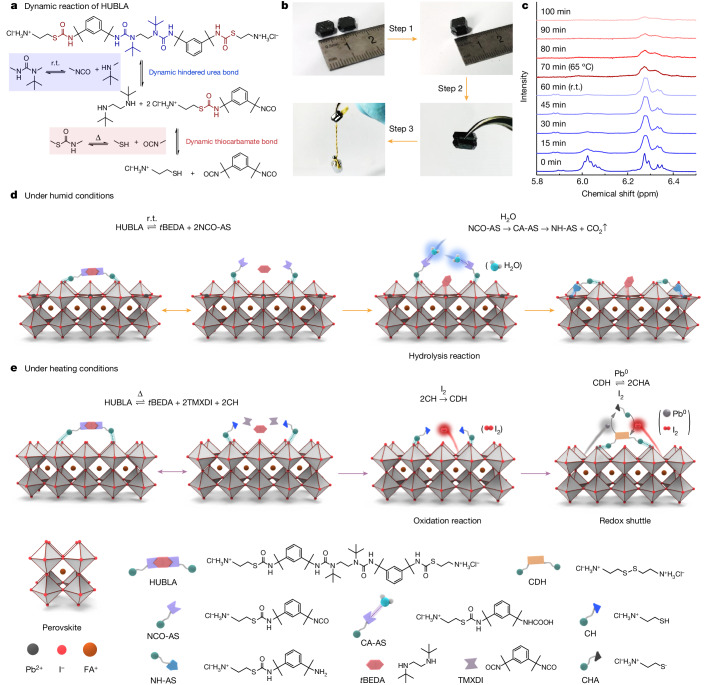
Dynamic reaction, hydrolysis and redox shuttle of HUBLA. **a**, Dynamic reactions of HUBLA. **b**, Photographs showing the connection of two FAPbI_3_ crystals using HUBLA. HUBLA in isopropanol (10 mg ml^−1^) was coated on one side of the FAPbI_3_ crystal, and another FAPbI_3_ crystal was placed on top of it for 24 h at room temperature. The two crystals showed a strong adhesion and could lift a 5-g weight. **c**, ^1^H nuclear magnetic resonance (NMR) characterizations of HUBLA in dimethyl sulfoxide-*d*_6_ and deuterium oxide (D_2_O) over 100 min. Immediately after the ^1^H NMR test at 0 min, 15% D_2_O was added; the temperature was then increased to 65 °C after 60 min. **d**, Dynamic and hydrolysis reactions of HUBLA coated on a perovskite film under humid conditions. **e**, Dynamic and redox reactions of HUBLA coated on a perovskite film under heating conditions. CH, cysteamine hydrochloride.

Accordingly, we designed and synthesized a hindered urea/thiocarbamate bond Lewis acid–base material (HUBLA) (Fig. 1a) as a living passivator by incorporating a HUB and a TCB, which can be triggered by environmental factors to generate new passivators. The characterization of the chemical structure of HUBLA is shown in Supplementary Fig. 4. Effectively, *N*,*N*′-di-*tert*-butylethylenediamine (*t*BEDA) can quench the –NCO group, preventing it from reacting with weak Lewis bases, such as isopropanol, as detailed in Supplementary Note 2 and Supplementary Fig. 5.

To evaluate the dynamic behaviour of HUBLA, Fig. 1b shows that it is possible to join two formamidinium lead iodide (FAPbI_3_) crystals together with HUBLA. The joined crystals showed a strong adhesion and could lift 5 g to 20 g of weight with different curing times. This shows the association–dissociation reaction and formation of a cross-linked structure of HUBLA between the two crystals, as further discussed in Supplementary Note 3, Supplementary Figs. 6–9, and Supplementary Table 1. On the basis of this discussion, we speculate that the strong ionic interactions of HUBLA could fill and protect the grain boundaries of perovskite against water penetration (Supplementary Fig. 10).

Following equation (2), the dynamic association–dissociation reaction mechanism indicates that HUBLA can reversibly dissociate into NCO-terminated ammonium salt (NCO-AS) and *t*BEDA at room temperature^6^ (Fig. 1a). In addition, according to equation (3), NCO-AS can undergo an association–dissociation reaction to generate cysteamine hydrochloride (CH) and 1,3-bis(1-isocyanato-1-methylethyl)benzene (TMXDI) at elevated temperatures^8^. Once in contact with water molecules, NCO-AS and TMXDI can decompose into NH_2_-terminated ammonium salt (NH-AS) and TMXDI hydrolysate, respectively^7^. To demonstrate this, ^1^H NMR spectra were obtained to verify the dissociation of HUB (–N(C(CH3)3)C(O)N(H)–) and TCB (–SC(O)N(H)–) from HUBLA. HUB reacted with deuterium oxide (D_2_O) at room temperature, whereas TCB reacted with D_2_O above 65 °C (Fig. 1c and Supplementary Note 4).

## Dynamic behaviour of HUBLA on perovskite

HUBLA can provide several strategies for the passivation of perovskite film. All the reaction mechanisms are described in Supplementary Fig. 11. As there were no changes in the surface morphology and crystallinity of the perovskite/HUBLA films (Supplementary Figs. 12 and 13), we investigated their optoelectronic properties. We measured photoluminescence (PL) and time-resolved photoluminescence (TRPL) spectroscopy for the pristine and HUBLA-coated perovskites. In Supplementary Fig. 14, the photoluminescence and time-resolved photoluminescence spectroscopy spectra show that the HUBLA-coated perovskite had a higher photoluminescence intensity with a longer lifetime. The enhanced photoluminescent performance of the perovskite can be attributed to the reduced trap density^22^. Non-hysteretic current–voltage (*J*–*V*) characteristics with (red line) and without (grey line) HUBLA (Supplementary Fig. 15 and Supplementary Note 5), obtained from pulsed-voltage space-charge-limited-current measurements^23^, showed a lower-bound trap density of 5.06 × 10^10^ cm^−3^ for the HUBLA-treated perovskite and 3.90 × 10^11^ cm^−3^ for the pristine perovskite. The substantial reduction in the lower-bound trap density for the HUBLA-treated perovskite indicates the successful passivation through HUBLA. In the following, we study the dynamic passivation through HUBLA. First, we used X-ray photoelectron spectroscopy (XPS) to understand the chemical evolution of the different compounds. Second, we measured photoluminescence maps to show the dynamic passivation.

**Fig. 2:**
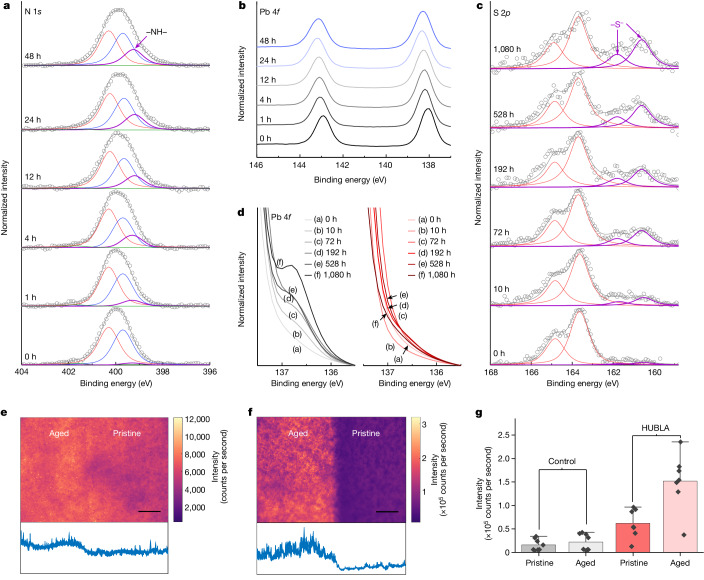
Dynamic reaction and passivation of HUBLA on perovskite films. **a**, N 1*s* XPS spectra of the films aged at 25 °C and 65% relative humidity. The red, blue, green and purple curves represent respective N 1*s* components of FA^+^, the amide bond (also including –NH_2_), ammonia and –NH–. **b**, Pb 4*f* XPS spectra of the films aged at 25 °C and 65% relative humidity. **c**, S 2*p* XPS spectra of the films aged at 85 °C in N_2_. The red and purple curves represent S 2*p* spectra of the TCB (also including –S–S–) and –S^−^, respectively. In **a** and **c**, the grey circles and curves represent the raw data and peak sum, respectively. **d**, Pb 4*f* XPS spectra of the films aged at 85 °C in N_2_. The left and right panels are perovskite (grey curves) and perovskite/HUBLA (red curves) films, respectively. **e**,**f**, Photoluminescence maps of perovskite films coated without (**e**) and with (**f**) HUBLA. Scale bar, 50 μm. The inset below shows a horizontal cross-section of the middle of the photoluminescence map. **g**, Statistical difference in photoluminescence for aged and pristine samples. Each point is an individually measured photoluminescence map. The bar indicates the mean and the error bar is 1.5 interquartile range. It is 8 control samples and 7 samples for HUBLA.

As mentioned shown equation (2), HUBLA can reversibly dissociate into *t*BEDA and NCO-AS at room temperature; the generated NCO-AS can react with a water molecule and then hydrolyse to NH-AS^7^ (Fig. 1d and Supplementary Fig. 11c). Both –NH– (from *t*BEDA) and –NH_2_ (from NH-AS) have the capability to heal ionic vacancies of the perovskite films^24^. We aged FAPbI_3_ with and without HUBLA under humid conditions (25 °C, 65% relative humidity) to study the hydrolysis reaction of HUBLA and measured the N 1*s* core-level spectra via XPS near the surface (Fig. 2a). The identification of the N 1*s* level is discussed in Supplementary Note 6, and Supplementary Figs. 16 and 17. Figure 2a shows that there are four components in the N 1*s* region of perovskite/HUBLA, assigned to FA^+^ (red curve), the amide bond (–N(R)C(O)–) in HUBLA (blue curve), the ammonia (green curve) and the –NH– group (purple curve) in *t*BEDA. With further ageing, the N 1*s* component of –NH– continuously increased for 48 h, indicating that the ambient moisture continuously reacts with the NCO-AS, causing HUBLA to constantly dissociate and produce *t*BEDA. We also verified the dynamic process via an attenuated total reflection infrared spectra of a perovskite/HUBLA film (Supplementary Fig. 18 and Supplementary Note 7), which showed a peak at 1,734 cm^−1^, representing the carbamic acid group (–N(H)C(O)OH) in carbamic acid group-terminated ammonium salt (CA-AS)^25^.

To understand the capability of HUBLA’s hydrolysates (*t*BEDA and NH-AS) for perovskite passivation under humid conditions, we conducted density functional theory (DFT) calculations and measured the Pb 4*f* XPS spectra of perovskite/HUBLA films at 25 °C and 65% relative humidity. Density functional theory calculations showed that *t*BEDA and NH-AS could interact with the positively charged undercoordinated Pb ions (Supplementary Figs. 19–21). In the Pb 4*f* XPS spectra (Fig. 2b), the Pb^2+^ peaks shifted towards higher binding energy during ageing, suggesting that there are more negative charges around Pb^2+^ ions^26,27^. To further demonstrate the passivation capability of –NH_2_ and –NH–, we coated TMXDI hydrolysate and *t*BEDA onto the perovskite films and measured their Pb 4*f* XPS spectra (Supplementary Fig. 22). The peaks of the perovskite/TMXDI hydrolysate and perovskite/*t*BEDA shifted towards higher binding energy, indicating that –NH_2_ and –NH– interact with the Pb^2+^ ion^24^. Moreover, we determined the threshold for the water-activated passivation through a 48-hour hydrolysis test of HUBLA at 25 °C and a relative humidity range from 20% to 90%, as shown in Supplementary Fig. 23. The results indicate that HUBLA can undergo hydrolysis even at 20% relative humidity.

In addition to the water-activated dynamic behaviour, we also measured the changes in the surface chemistry of HUBLA coated on perovskite films during thermal ageing (Fig. 1e). In general, lead iodide-based perovskites degrade into PbI_2_ and then generate I_2_ and Pb^0^ under thermal stress or light soaking^11,28^. The thiol group (–SH) in cysteamine hydrochloride, dissociated from the TCB at elevated temperature (equation (3)), can be oxidized by I_2_ and produce disulfide-containing (–S–S–) cystamine dihydrochloride (CDH). The disulfide bond can be further reduced by Pb^0^ and produce a sulfur-anion (–S^−^)-containing cysteamine hydrochloride anion (CHA). Here, CDH and CHA can act as redox shuttles to selectively and cyclically oxidize Pb^0^ and reduce I_2_ (detailed reactions in Supplementary Fig. 11d). To verify the hypothesis, we aged a perovskite/HUBLA film at 85 °C in nitrogen (N_2_) and measured the S 2*p* core-level spectra. The identification of the S 2*p* XPS spectra of the samples is discussed in detail in Supplementary Note 8 and Supplementary Fig. 24. In particular, the S 2*p* region of perovskite/HUBLA films (Fig. 2c) showed two components, assigned to the TCB (–SC(O)N(H)–) in HUBLA (red curve) and the sulfur anion (–S^−^) in CHA (purple curve). CHA increased with thermal ageing from 0 h to 1,080 h, indicating the continuous dissociation of HUBLA and the reduction reaction of CDH. To directly observe the redox reaction, we showed the colour change of an I_2_ solution and the dissolution of a lead pellet through the addition of HUBLA (Supplementary Figs. 25 and 26, and Supplementary Note 9). Furthermore, we measured the standard electrode potential for the redox reaction (Supplementary Fig. 27). Upon analysing the redox peaks present in the cyclic voltammetry graph, we found that the standard electrode potential for –S^−^·*x*PbI_2_/–S–S–·*x*PbI_2_ was 0.487 V. This potential lies within the range of PbI_2_/Pb (−0.365 V) and I_2_/I^−^ (0.536 V), indicating that the redox reactions are thermodynamically feasible^29^.

To understand the capability of the redox shuttles (CDH and CHA) for perovskite passivation under heating conditions, we measured the Pb 4*f* XPS spectra of perovskite and perovskite/HUBLA films aged at 85 °C in N_2_, as shown in Supplementary Fig. 28. The Pb^2+^ showed two peaks at around 138.3 eV and around 143.2 eV, and Pb^0^ showed two peaks at around 136.7 eV and around 141.6 eV. As shown in Fig. 2d and Supplementary Fig. 28, the ratio of Pb^0^/(Pb^0^ + Pb^2+^) in the perovskite film increased from 0.2% to 3.7% after heating for 1,080 h, whereas the ratio in the perovskite/HUBLA film remained relatively low (from <0.001% to 0.3%) under the same conditions, which can be attributed to the redox reactions of –SH (from cysteamine hydrochloride), –S–S– (from CDH) and –S^−^ (form CHA). We found the threshold for the heat-activated passivation of HUBLA to be ≥55 °C (Supplementary Fig. 29).

To observe the dynamic passivation of HUBLA on perovskite films, photoluminescence spectroscopy maps of the perovskite films were measured during ageing. We exposed a small area of the film to intense light (96.5 suns) for 2 h. After ageing, we left the samples for 10 min in the dark and measured a photoluminescence map with half of the area aged and the other half not exposed to light during the ageing, which we refer to as pristine. We observed a slight improvement in photoluminescence intensity in the aged control area compared with the pristine area. This photo brightening is a relatively well-described phenomenon in lead halide perovskites and has been previously studied^30^. However, compared with the control, all HUBLA films showed substantially stronger improvements in photoluminescence, around one order of magnitude larger in counts per second. We present an example of two films (control and HUBLA) in Fig. 2e,f with their corresponding histograms provided in Supplementary Fig. 30. A statistical analysis of all the samples is presented in Fig. 2g. Given that photoluminescence is a direct measure of the amount of radiative recombination, the ability of HUBLA to comparatively increase the photoluminescence significantly more during ageing indicates that HUBLA is dynamically passivating defects or traps in the photo absorber, and healing the perovskite film.

## Stability of HUBLA-coated perovskite

**Fig. 3:**
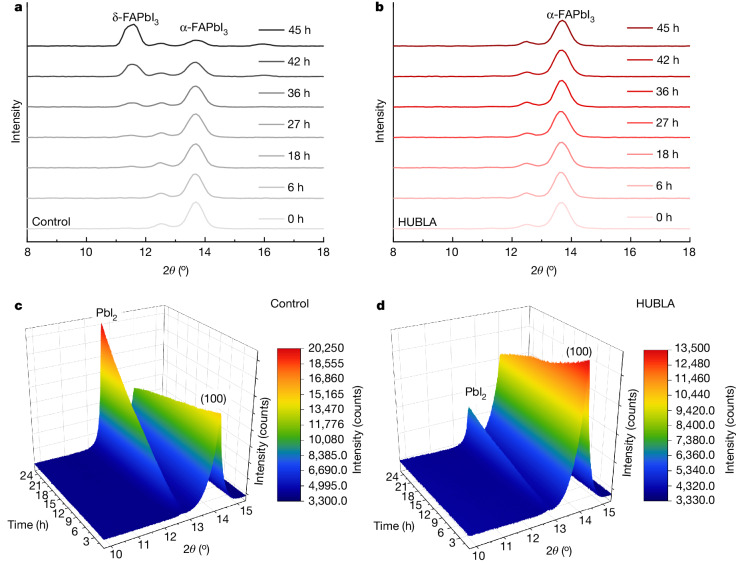
Stability of perovskite films. **a**,**b**, XRD patterns of perovskite films without (**a**) and with (**b**) HUBLA aged at 25 °C and 60–70% relative humidity. 2θ is the angle between the incident and the diffracted X-ray beam directions. **c**,**d**, In situ XRD^2^ patterns of perovskite films without (**c**) and with (**d**) HUBLA heated at 85 °C under 30% relative humidity. Initial and final XRD^2^ measurements are shown in Supplementary Fig. 33 and the change along the azimuthal angle *χ* for the PbI_2_ peaks is depicted in Supplementary Fig. 34a,b. The ratio of the peak area of (100) and PbI_2_ peak over time is presented in Supplementary Fig. 34c.

In the following, we investigate the impact of HUBLA on the stability of perovskite films under humid and heating conditions. A moisture stability test was performed on FAPbI_3_ films stored at 25 °C and 60–70% relative humidity (Supplementary Fig. 31). The surface of the pristine perovskite film began to turn yellow after 18 h, which was caused by the phase transformation to δ-FAPbI_3_. In comparison, the HUBLA-coated perovskite remained black after 45 h. The X-ray diffraction (XRD) patterns (Fig. 3a,b) and their corresponding δ-phase/α-phase ratios (Supplementary Fig. 32) showed that the δ-phase perovskite in the pristine perovskite film became noticeable after 36 h, but not in the HUBLA-treated sample.

To test the thermal stability, we decided to use a FA_0.83_Cs_0.17_Pb(I_0.9_Br_0.1_)_3_ perovskite as it has been shown to have superior stability under light and heat^3,31^. For the commercialization of PSCs, it is necessary to pass the IEC 61215, the international standard for PSCs^32^. The highest used temperature in the IEC 61215 is 85 °C. Therefore, we measured in situ two-dimensional XRD (XRD^2^) of two perovskite thin films by heating at 85 °C under 30% relative humidity ambient condition for more than 24 h (Fig. 3c,d and Supplementary Fig. 33). The pristine perovskite showed an increase in PbI_2_ (the degradation product of the perovskite film^33,34^) throughout ageing. In comparison, the thin film treated with HUBLA developed roughly two-thirds less PbI_2_, as discussed in Supplementary Note 10 and Supplementary Figs. 34–37).

## Device performance and stability

In the following, we demonstrate that the HUBLA passivation works for n–i–p and p–i–n devices. We fabricated n–i–p devices using a stack of ITO/SnO_2_/FA_0.72_MA_0.28_PbI_3_/without and with (wo/w) HUBLA/spiro-OMeTAD/MoO_3_/Ag. The highest achieved PCE for HUBLA devices was 24.7%, with a short-circuit current density (*J*_SC_) of 25.6 mA cm^−2^, an open-circuit voltage (*V*_OC_) of 1.16 V and a fill factor of 0.83; for the control device, we reached 21.7% with a *J*_SC_ of 25.6 mA cm^−2^, a *V*_OC_ of 1.10 V and a fill factor of 0.77 (Supplementary Fig. 38). As seen in the inset of Supplementary Fig. 38a, stabilized power output (SPO) tracking for 30 s showed that the HUBLA cell had a steady-state efficiency of 24.5%, whereas that of the control cell was 21.3%. In addition, the external quantum efficiency (EQE) of the HUBLA device showed an integrated current density of 24.4 mA cm^−2^ (Supplementary Fig. 39), and the total absorbance spectrum of glass/ITO/perovskite is shown in Supplementary Fig. 40. In Supplementary Fig. 38b, we measured the stability of the n–i–p devices under ambient condition with 25 °C and 30% relative humidity. The normalized PCE showed that the HUBLA devices retained nearly 87% of the original performance, whereas that of the control devices dropped to 43% after 3,600 h. It is noted that the PCE of the HUBLA devices only slightly decreased before 768 h, whereas that of the control devices decreased significantly after 336 h, which is discussed in Supplementary Note 11 and Supplementary Fig. 41.

**Fig. 4:**
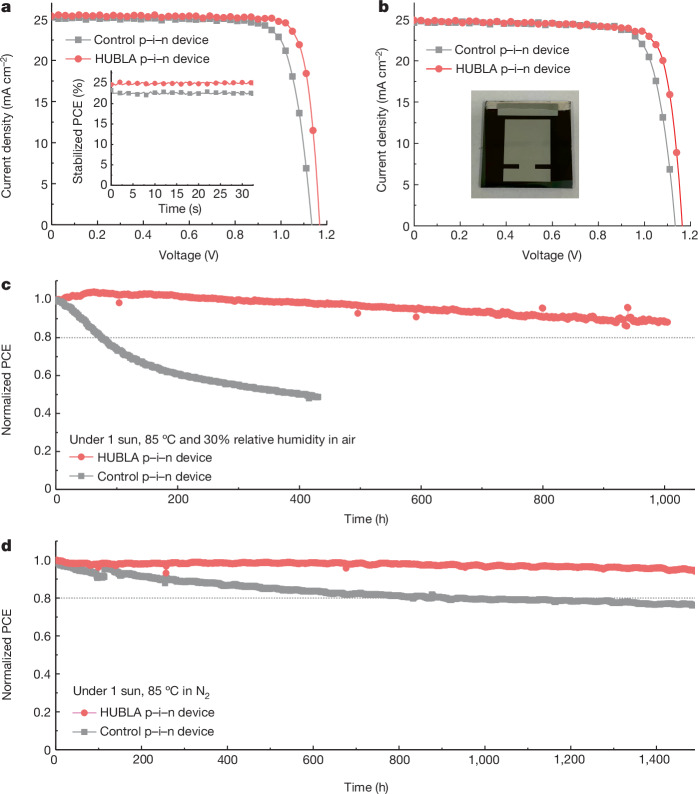
Performance and stability of perovskite photovoltaics. **a**, Best *J*–*V* curves with SPO of p–i–n devices. **b**, Best *J*–*V* curves of 1-cm^2^ p–i–n devices (the same device structure as in **a**). The inset shows the photograph of 1-cm^2^ device. **c**, The MPPT tests of encapsulated p–i–n devices (the same device structure as in **a**) aged at 85 °C and 30% relative humidity in air under 1 sun. The initial PCEs of the control and HUBLA devices were 20.9% and 23.0%, respectively. **d**, The MPPT tests of unencapsulated p–i–n devices aged at 85 °C in N_2_ under 1 sun. The initial PCEs of the control and HUBLA devices were 20.8% and 23.3%, respectively. The horizontal dashed line represents 80% of initial PCE value.

It is known that p–i–n devices show better thermal stability compared with n–i–p devices owing to the absence of a doped organic charge transport layer on top of the perovskite. Therefore, we fabricated p–i–n devices with the device architecture of ITO/2PACz/FA_0.72_MA_0.18_Cs_0.1_PbI_3_/(wo/w) HUBLA/C_60_/bathocuproine/Au), demonstrating a best PCE of 25.1% for the HUBLA device with a *V*_OC_ of 1.17 V, a *J*_SC_ of 25.4 mA cm^−2^ and a fill factor of 0.84. However, the control device showed a PCE of 22.7% with a *V*_OC_ of 1.13 V, a *J*_SC_ of 25.2 mA cm^−2^ and a fill factor of 0.79 (Fig. 4a). As seen in the inset of Fig. 4a, SPO tracking for 30 s showed that the HUBLA cell had a steady-state efficiency of 25.0%, whereas that of the control cell was 22.5%. Moreover, we fabricated the p–i–n device with an effective area of 1 cm^2^ and achieved up to 23.5% for the HUBLA devices (Fig. 4b), whereas that of the control device was 22.2%. To assess the capability of HUBLA in enhancing device stability under thermal and humid conditions, we performed the maximum power point tracking (MPPT) of encapsulated control and HUBLA devices under 85 °C and about 30% relative humidity in air (ISOS-L-2) (Fig. 4c). It was observed that the control device rapidly declined to below 50% after 431 h, whereas the HUBLA device retained 88% of its initial value after 1,000 h. Accordingly, we investigated the hydrolysis reaction of HUBLA at elevated temperature, and performed N 1*s* XPS analysis on the perovskite/(wo/w) HUBLA film under 85 °C and 30% relative humidity (Supplementary Fig. 42). This analysis showed that the intensity of the –NH– group continuously increased at 85 °C under 30% relative humidity, indicating that water-activated passivation can also occur at high operating temperatures under humid conditions.

We also studied the impact of HUBLA on devices under 85 °C in the dark and in N_2_ (ISOS-D-2). In median, the HUBLA devices retained 90% of their initial efficiency after 21,864 h (Supplementary Note 12 and Supplementary Figs. 43–45). In addition, we fabricated a HUBLA-based FA_0.92_Cs_0.08_PbI_3_ p–i–n device and obtained a certified PCE of 24.3% (Supplementary Fig. 46), and performed the MPPT under 85 °C in N_2_ without encapsulation (ISOS-L-2) (Fig. 4d). The time required to drop to 80% of the initial efficiency (*T*_80_) of the control device was recorded to be 866 h, whereas the HUBLA device maintained 94% of the initial PCE after ageing near 1,500 h. Considering the S 2*p* XPS spectra (Fig. 2c), which show the dissociation of the TCB in HUBLA and the formation of a redox shuttle to reduce I_2_ and oxidize Pb^0^ (Fig. 2d), it can be inferred that the enhanced thermal stability is attributed to the ability of HUBLA to inhibit perovskite degradation and to extend operational and storage stability.

In summary, we propose a real-time responsive passivation strategy for perovskites using HUBLA with DCBs, which can passivate as-deposited perovskite films not only during the fabrication process but also afterwards. With HUBLA and its evolving products, we have achieved enhanced device performance and realized improved operational stability at elevated temperatures and under humid conditions.

## Methods

### Materials

Tin(iv) oxide (SnO_2_; 15% in H_2_O colloidal dispersion), lead(ii) iodide (PbI_2_; 99.999%), cesium iodide (CsI; 99.999%), molybdenum(vi) oxide (MoO_3_), isopropanol (IPA; 99.5%), diethyl ether (99%), *N*,*N*-dimethylformamide (DMF; 99.8%), dimethyl sulfoxide (DMSO; 99.9%), chlorobenzene (CB; 99%), acetonitrile (ACN; 99.8%) and bathocuproine (BCP) were purchased from Thermo Scientific Chemicals. Triethanolamine (TEA; 99%), γ-butyrolactone (GBL; 99%), triethylene glycol (TEG; 99%), *N*,*N*′-di-*tert*-butylethylenediamine (*t*BEDA; 98%), 1,3-bis(1-isocyanato-1-methylethyl)benzene (TMXDI; 97%), cysteamine hydrochloride (CH; 98%), lithium bis(trifluoromethanesulfonyl)imide (LiTFSI; 99.99%), 4-*tert*-butylpyridine (tBP; 98%), anisole (anhydrous, 99.7%), dimethyl sulfoxide-*d*_6_ (DMSO-*d*_6_), deuterium oxide (D_2_O) and dibutyltin dilaurate (DBTDL; 95%) were purchased from Sigma-Aldrich. Formamidinium iodide (FAI) and methylammonium iodide (MAI) were purchased from Greatcell Solar Materials Pty Ltd. Lead(ii) chloride (PbCl_2_) and [6,6]-phenyl-C_61_-butyric acid methyl ester (PCBM) were purchased from TCI America. 2,2′,7,7′-Tetrakis-(*N*,*N*-di-4-methoxyphenylamino)-9,9′-spirobifluorene (spiro-OMeTAD; 99.5%), 2,3,5,6-tetrafluoro-7,7,8,8-tetracyano-quinodimethane (F4-TCNQ) and fullerene (C_60_) were purchased from Luminescence technology corp. Gold (Au) and silver (Ag) pellets were purchased from Kurt J. Lesker Company. Poly(4-butylphenyl-diphenyl-amine) (Poly-TPD) was purchased from 1-Material. [2-(9*H*-Carbazol-9-yl)ethyl]phosphonic acid (2PACz) was purchased from Xi’an Yuri Solar Co., Ltd. (4-(7*H*-dibenzo[*c*,*g*]carbazol-7-yl)butyl)phosphonic acid (CbzNaph) was prepared according to the previous literature^35^. HelioSeal PVS101 Tape was obtained from Koemmerling (Nanjing) Advanced Material Co., Ltd. UV curable resin (TB3035B) was obtained from ThreeBond. Indium tin oxide (ITO) glasses (with and without antireflection coating) were purchased from South China Science & Technology Company Limited.

### Preparation of HUBLA

First, 1.72 g *t*BEDA (10 mmol) and 10 ml DMF were charged in a 250-ml three-necked flask under an inert N_2_ atmosphere and kept in an ice bath. Then 4.89 g TMXDI (20 mmol) was dropped into the solution and stirred for 2 h. Afterwards, 2.27 g cysteamine hydrochloride (20 mmol) and 3 drops of DBTDL were dissolved in 20 ml DMF and then dropped in the reaction system. The reaction was carried out for 3 days and then poured into dried diethyl ether. The precipitates were collected and purified by precipitation in dried diethyl ether three times, and the white solid was then dried under vacuum overnight.

### Preparation of poly(urea-urethane) elastomer and self-healing experiment

In a 50-ml three-necked flask, 3.71 g TMXDI (15.2 mmol) was dissolved in 5 ml DMF and cooled to 4 °C. Then 0.86 g *t*BEDA (5 mmol) was slowly dropped into the above solution to form oligo-urea. Afterwards, 0.188 g TEA (1.26 mmol), 1.282 g TEG (8.54 mmol) and 2 drops of DBTDL were added and the solution was vigorously homogenized. Finally, the polymer solution was charged into a 5 × 5 × 0.5 cm^3^ mould followed by curing at room temperature for 24 h under vacuum to remove DMF. For the self-healing test of elastomer, 14 black cubes and 13 white cubes were stuck together and self-healed for 24 h at 25 °C.

### Preparation of single-crystal FAPbI_3_

The FAPbI_3_ single crystals were fabricated from a seed crystal by following a previously published protocol^36,37^. The control FAPbI_3_ single crystals were prepared by dissolving equimolar FAI and PbI_2_ in γ-butyrolactone. The solution was stirred at 60 °C for 1 h and then filtered with a 25-mm-diameter 0.45-µm glass microfibre filter. Then 4 ml of the filtrate was placed in a vial that contained a seed FAPbI_3_ crystal, and the vial was kept in an oil bath undisturbed at 95 °C for 8 h. To control the thickness, a small chamber was constructed using two thin glass plates placed on a glass plate at the bottom of the vial with a gap in between and a cover glass on top. Free-standing and millimetre-sized crystals were removed from the vial once formed. For the HUBLA treatment, the crystals were dipped into the HUBLA solution. For testing the bonding forces with different curing times, the surface of the crystal was washed with dried IPA in a N_2_ glovebox. For the crystal adhesion test, HUBLA was coated on one side of a FAPbI_3_ crystal, and another FAPbI_3_ crystal was then placed on top of it and cured at room temperature for different times.

### Fabrication of FA_0.72_MA_0.28_PbI_3_ composition n–i–p PSCs

#### Substrate preparation

ITO-coated glass substrate was sequentially sonicated in deionized water, acetone and IPA, then treated with ultraviolet-ozone for 10 min after being dried with an air gun.

#### SnO_2_ layer preparation

SnO_2_ nanoparticles (2.67%, diluted by deionized water) were spun onto the above ITO substrate at 5,000 rpm for 30 s, and then annealed in ambient air at 150 °C for 30 min.

#### FA_0.72_MA_0.28_PbI_3_ composition

A 1.3 M FA_0.72_MA_0.28_PbI_3_ precursor solution was prepared by dissolving 553 mg PbI_2_, 58 mg MAI and 160 mg FAI into a 1 ml mixed solvent of DMF:DMSO (v:v = 9:1), and 27 mg PbCl_2_ was also added as an additive. The perovskite precursor solution was deposited onto the prepared substrate at 1,000 rpm for 10 s and 5,000 rpm for 20 s. During the spin-coating step, 100 µl chlorobenzene was poured on the precursor film at 10 s before the end of spin and the film was then annealed at 100 °C for 30 min in a N_2_ glovebox^38^. For the HUBLA device, HUBLA (0.03–0.2 mg ml^−1^ in IPA) was spun onto the perovskite film.

#### Hole transport layer and electrode deposition

Spiro-OMeTAD was dissolved in 1 ml of chlorobenzene solution, which contained 72.3 mg spiro-OMeTAD, 18 µl lithium bis(trifluoromethanesulfonyl)imide solution (520 mg ml^−1^ in acetonitrile) and 30 µl 4-*tert*-butylpyridine. Hole-transporting material was deposited onto the perovskite film at a spin rate of 4,000 rpm for 30 s. The above film was then left overnight under controlled ambient conditions. Finally, MoO_3_ was evaporated onto the samples under vacuum of <10^−5^ torr at a rate of 0.1–0.2 Å s^−1^, and then 100-nm Ag was evaporated under vacuum of <10^−5^ torr at a rate of 1–2 Å s^−1^.

### Fabrication of FA_0.72_MA_0.18_Cs_0.1_PbI_3_ composition p–i–n PSCs

#### Self-assembled-monolayer preparation

2PACz solution was spin-coated on ITO substrate at 4,000 rpm for 30 s and then annealed at 120 °C for 10 min.

#### FA_0.72_MA_0.18_Cs_0.1_PbI_3_ composition

A 1.3 M perovskite precursor solution (1 ml) was prepared by mixing FAI, PbI_2_, MAI and CsI in DMF:DMSO mixed solvent (9:1, v:v) with the chemical formula FA_0.72_MA_0.18_Cs_0.1_PbI_3_, and 27 mg PbCl_2_ was also added as the additive. The fabrication of the perovskite and HUBLA was the same as the procedure of fabricating FA_0.72_MA_0.28_PbI_3_ composition n–i–p PSC.

#### Electron transport layer and electrode deposition

Afterwards, C_60_ and BCP were evaporated onto the samples under vacuum of <5 × 10^−6^ torr at a rate of 0.1–0.3 Å s^−1^. Finally, 100-nm Au electrode was deposited by a thermal evaporator at the rate of 1–2 Å s^−1^.

### Fabrication of FA_0.92_Cs_0.08_PbI_3_ composition p–i–n PSCs

#### Self-assembled-monolayer preparation

Cleaned ITO substrates were treated with ultraviolet-ozone for 30 min and transferred to a N_2_ glovebox for film fabrication. CbzNaph (0.5 mg ml^–1^ in IPA) was spin-coated onto the cleaned ITO at 3,000 rpm for 30 s, followed by annealing at 100 °C for 15 min.

#### FA_0.92_Cs_0.08_PbI_3_ composition

A 1.7 M perovskite precursor solution with the chemical formula FA_0.92_Cs_0.08_PbI_3_, 5% excess PbI_2_ and 10 mol% MACl were dissolved in a mixed solvent of DMF:DMSO (v:v = 4:1). Then, 50 μl of the prepared precursor solution was spin-coated onto the self-assembled-monolayer-based ITO substrate at 1,000 rpm for 10 s and 5,000 rpm for 40 s. At 10 s before the end of the last procedure, 180 μl chlorobenzene was dripped onto the film. Afterwards, the substrate was annealed at 100 °C for 30 min. After deposition of the perovskite active layer, 50 μl HUBLA (dissolved in IPA) solution was spin-coated onto the film.

#### Electron transport layer and electrode deposition

PCBM (5 mg ml^−1^) was spin-coated onto the perovskite film and annealed at 70 °C for 10 min. The films were then cooled to room temperature and readied for thermal evaporation. Subsequently, 25-nm C_60_, 8-nm BCP and 100-nm Au electrode were sequentially evaporated under vacuum of <5 × 10^−6^ torr.

### Fabrication of FA_0.83_Cs_0.17_Pb(I_0.9_Br_0.1_)_3_ composition p–i–n PSCs

#### Substrate cleaning

The fluorine doped tin oxide (FTO) substrates (30 × 30 mm^2^) were brushed with a detergent (Fairy Liquid) and subsequently sonicated for at least 10 min in a 4% v/v solution of Decon 90, acetone and IPA.

#### Poly-TPD layer preparation and deposition

First, 1 mg ml^−1^ poly-TPD was dissolved in an amber vial in toluene with 0.2 mg ml^−1^ F4-TCNQ. The solution was stirred overnight at 80 °C in a N_2_ glovebox. Before use, the solution was filtered with a 0.22-µm PTFE filter. The FTO substrates were treated with ultraviolet-ozone for 10 min directly before use. In ambient condition (30–55% relative humidity), 80 μl of poly-TPD was dynamically deposited onto the FTO substrate at 2,000 rpm (the acceleration of 2,000 rpm s^–1^) for 20 s. Afterwards, the substrates were heated for 5 min at 130 °C in ambient condition.

#### FA_0.83_Cs_0.17_Pb(I_0.9_Br_0.1_)_3_ perovskite and deposition

For preparing 1.45 M FA_0.83_Cs_0.17_Pb(I_0.9_Br_0.1_)_3_ perovskite precursor, 827.86 mg FAI, 256.17 mg CsI, 2,272.78 mg PbI_2_ and 319.30 mg PbBr_2_ were dissolved in 4 ml mixed solvent of DMF:DMSO (v:v = 4:1), followed by stirring at room temperature in the glovebox overnight before deposition. Before use, the solution was filtered with a 0.4-µm PTFE filter. For the first step of spin-coating, 200 μl solution was dynamically applied onto the substrate for 7–8 s. The spin-coating programme was 10 s at 1,000 rpm (the acceleration of 200 rpm s^–1^) and 35 s at 5,000 rpm (the acceleration of 2,000 rpm s^–1^). Ten seconds before the end of the second spin-coating step, 400 μl anisole was applied. The perovskite layer was annealed at 100 °C. Spin-coating and heating for the devices were done in a N_2_ glovebox.

#### PCBM, BCP layers and electrode deposition

First, 20 mg ml^−1^ PCBM was dissolved in a mixed solvent of chlorobenzene:1,2-dichlorobenzene (v:v = 3:1) overnight. Before use, the solution was filtered with a 0.22-μm PTFE filter. In a N_2_ glovebox, 50 μl PCBM was dynamically deposited onto the substrate at 2,000 rpm (the acceleration of 2,000 rpm s^–1^) for 20 s. The substrates were annealed afterwards for 4 min at 100 °C. Then 0.5 mg ml^−1^ BCP was dissolved in IPA and stirred overnight at 80 °C. Before use, the solution was filtered with a 0.22-µm PTFE filter. Seventy microlitres of BCP was deposited dynamically at 5,000 rpm (the acceleration of 2,000 rpm s^–1^) for 30 s. The substrates were finally annealed for 1 min at 100 °C. Finally, 75- to 100-nm Au was deposited through a shadow mask at <3 × 10^−6^ torr using a thermal evaporator (Nano 36, Kurt J. Lesker).

### Photovoltaic performance characterization

The FA_0.72_MA_0.28_PbI_3_ composition n–i–p devices and FA_0.72_MA_0.18_Cs_0.1_PbI_3_ composition p–i–n devices were measured with a Keithley 2400 source meter under a simulated AM 1.5G spectrum, and the active areas were 0.1 cm^2^ and 1 cm^2^ (0.1 cm^2^ and 1 cm^2^ defined using black masks in direct contact with the glass side of the substrates). With a solar simulator (Enli Technology), the light intensity was calibrated using a KG5 reference cell before each measurement and the scan rate of *J*–*V* curves was 200 mV s^−1^. External quantum efficiency measurements was carried out with a QE-R system (Enli Technology) at 25 °C and 30–45% relative humidity. Ageing tests of unencapsulated n–i–p devices were performed at 25 °C and 30% relative humidity. MPPT tests of the encapsulated p–i–n devices were measured at 85 °C and 30% relative humidity in air. The light source for MPPT tests was a BBZM-III xenon lamp with a filter. The MPPT tests of the unencapsulated p–i–n devices based on FA_0.92_Cs_0.08_PbI_3_ composition were subjected to testing within the high-throughput solar-cell lifetime test system developed by CRYSCO at 85 °C.

The FA_0.83_Cs_0.17_Pb(I_0.9_Br_0.1_)_3_ p–i–n devices were characterized in ambient conditions with the room temperature at 20–24 °C and 45% relative humidity under AM 1.5G simulated sunlight generated by a class AAA WaveLabs Sinus-220 solar simulator, using a Keithley 2400 source meter. The intensity of the solar simulator was set to produce 100 mW cm^−2^ equivalent irradiance using a certified KG3-filtered silicon reference photodiode (Fraunhofer ISE). The voltage was swept at a rate of 0.61 V s^−1^, first from forward bias to reverse bias (forward sweep) followed by a reverse sweep in the opposite scan direction. The minimum voltage was −0.1 V and the maximum voltage was 1.2 V. The areas were defined using black anodized aluminium shadow masks (0.25 cm^2^) in direct contact with the glass side of the substrates within enclosed sample holders. Ageing tests of unencapsulated p–i–n devices were performed at 85 °C in N_2_ over 2 years. The light source was a ViparSpectra V450 450W full-spectrum light-emitting-diode grow light. The intensity was measured with a certified KG3-filtered silicon reference photodiode (Fraunhofer ISE). The spectrum was collected using a fibre-coupled spectrometer (Ocean Optics MayaPro).

### Nuclear magnetic resonance characterization

The ^1^H NMR spectra of HUBLA were characterized with a Bruker Avance 400-MHz NMR spectrometer. The HUBLA sample was tested by dissolving in DMSO-*d*_6_ solvent. For the hydrolysis of HUBLA, the HUBLA sample was prepared by dissolving in DMSO-*d*_6_ first, and then D_2_O was added with a volume ratio of 15% after the spectrum at 0 min was obtained. The NMR tube was heated at 65 °C after 1 h. To record the generation of disulfide bond, cysteamine hydrochloride and iodine (I_2_) pellets were added into 10 ml D_2_O solution and stirred.

### Cyclic voltammetry characterization

Cyclic voltammetry measurements were performed utilizing a standard three-electrode set-up at a scan rate of 50 mV s^−1^ (CHI650D). Solid platinum electrodes were used as counter and working electrodes, and 0.1 M tetrabutylammonium hexafluorophosphate solution was used as electrolyte. Ag/AgCl (with the standard electrode potential of 0.199 V) was used as a quasi-reference electrode (PINE research).

### Attenuated total reflection infrared spectroscopy characterization

The attenuated total reflection infrared spectra were acquired using a Thermo Scientific Nicolet 6700 FTIR spectrometer, equipped with a diamond attenuated total reflection (ATR) crystal. The ITO/SnO_2_/perovskite and ITO/SnO_2_/perovskite/HUBLA samples were measured in ATR mode using a spectral range from 4,000 cm^−1^ to 400 cm^−1^ and signal average over 32 scans. For the attenuated total reflection infrared test, ITO/SnO_2_/perovskite and ITO/SnO_2_/perovskite/HUBLA films were aged at 25 °C and 30–40% relative humidity for 12 days. HUBLA solution was dropped on the perovskite film and dried by N_2_ flow.

### Steady-state photoluminescence spectroscopy and time-resolved photoluminescence characterizations

The photoluminescence spectra of FAPbI_3_ and HUBLA-coated FAPbI_3_ single crystals were recorded using a photoluminescence spectrometer (FLS1000) with an excitation wavelength of 532 nm. The time-resolved photoluminescence spectroscopy spectra of FAPbI_3_ and HUBLA-coated FAPbI_3_ crystals were obtained using the FLS1000 with an excitation wavelength of 405 nm. For the photoluminescence and time-resolved photoluminescence spectroscopy tests of HUBLA-coated FAPbI_3_ crystals, FAPbI_3_ crystals were dipped into 0.125 mg ml^−1^ HUBLA solution and blown dry with N_2_ flow.

### Scanning electron microscopy characterization

The scanning electron microscopy (SEM) images of perovskite films were obtained with a Hitachi S-4800 operated at 15 kV.

### X-ray diffraction characterization

The XRD analysis was carried out using an X-ray powder diffractometer (D8 Discover, Brucker) with Cu Kα (wavelength*,* 1.54059 Å) radiation. For the ageing tests, FAPbI_3_ and FAPbI_3_/HUBLA films were tested at 25 °C and 60–70% relative humidity.

In situ XRD^2^ patterns were acquired using a Rigaku SmartLab X-ray diffractometer with a Cu Kα1 and a HyPix-3000 two-dimensional hybrid pixel array detector, operated at 40 kV. Two-dimensional one-shot XRD patterns were measured every minute, from 9.75° to 15.36° without moving the two-dimensional detector. Each two-dimensional pattern was folded to generate the plotted one-dimensional XRD. An Anton Paar heating stage was used together with the Rigaku SmartLab diffractometer for heating. XRD^2^ is the name of a two-dimensional XRD measurement that measures not only the 2*θ* direction but also the conic section of a polycrystalline sample. The diffraction compared with a normal single crystal is not an individual peak but a diffraction cone. Measuring it with a two-dimensional detector results in the appearance of characteristic diffraction rings, showing the spread of the diffraction peak in γ-direction. More information about XRD^2^ can be found in ref. ^39^. For the in situ XRD^2^, FA_0.83_Cs_0.17_Pb(I_0.9_Br_0.1_)_3_ wo/w HUBLA were aged at 85 °C at 30% relative humidity ambient condition.

### In situ photoluminescence

For the photoluminescence map measurements, the thin-film samples were prepared by the precursor solution using 1.6 M FAPbI_3_:MACl in a mixed solvent of DMF:DMSO (v:v = 4:1) (for example, 633 mg of FAPbI_3_ and 23.6 mg of MACl in 500 µl DMF and 125 µl DMSO), and were deposited on FTO glasses. We statically spread 160 µl perovskite precursor solution on the FTO substrate and started the spin-coating programme (6,000 rpm, 3,000 rpm s^−1^ acceleration) for 50 s. At 10–12 s, we quenched with 250 µl of anisole. Afterwards, we annealed the films at 150 °C for 15 min. The perovskite-layer fabrication was performed in a N_2_ glovebox.

The thin-film sample was mounted in a Nikon Ti2 microscope base with a 130-W mercury lamp, Kymera 328i spectrometer, and Zyla 4.2 PLUS scientific complementary metal–oxide–semiconductor. The light was monochromated using a Semrock brightline filter cube to provide a 488-nm excitation. The light intensity was 9.65 W cm^−2^ at ×60 magnification. The illuminated area was 4.91 × 10^−4^ cm^2^. The photoluminescence signal was normalized by the exposure time to ensure comparability between the images of different films. The samples were mounted with the surface towards the objective.

### Pulsed-voltage space-charge-limited-current measurement

For electrical characterization, 120-nm Au electrodes were evaporated on either side of the single crystals to obtain symmetric devices. Au electrodes were evaporated on both of the larger faces of the single crystal by an evaporator (Kurt J. Lesker, Nano 36) at a 0.5 Å s^−1^ deposition rate. The *JV* traces were measured using a computer-controlled 2400 Series Keithley source meter in the dark under vacuum at room temperature^35^. The vacuum pump (Leybold vacuum, PT 70 F-Compact) pumped the system down to 10^−4^ mbar.

### Optical microscopy

Optical microscopy images were taken on a Nikon Eclipse LV100ND microscope with Nikon TU Plan Fluor lenses (×10/0.30 A, ×20/0.45 A, ×50/0.60 B, ×100/0.90 A). The images were taken with an attached Nikon Digital Camera D6.10.

### Ultraviolet–visible absorption spectroscopy

Absorbance spectra of perovskite films were obtained with a Varian Cary 300 Bio ultraviolet–visible spectrophotometer with a 50 × 50 mm^2^ reflective neutral density filter with an optical density of 3.0 (made out of ultraviolet-fused silica). Total absorbance (1 − reflectance) was conducted on UV-3600 plus (SHIMADZU).

### X-ray photoelectron spectroscopy

XPS spectra were acquired on a Thermo Fisher K-Alpha system (X-ray source, Al Kα; pressure, 1 × 10^−8^ Pa). For the ageing tests, FAPbI_3_ and FAPbI_3_/HUBLA films were aged under 20–90% relative humidity within an Arc Test chamber (Xi’an LIB Environmental Simulation Industry) for N 1*s* XPS, and FAPbI_3_ and FAPbI_3_/HUBLA films were aged at 25–95 °C within an Arc Test chamber or N_2_ glovebox for S 2*p* XPS.

### Theoretical calculation

The spin theoretical simulations were performed on the Vienna Ab initio Simulation Package (version 5.4.1)^40^. In addition, electron–electron exchange and correlation interactions were evaluated by the generalized gradient approximation with the Perdew–Burke–Emzerhof^41^ functional form. The core–electron (valence electron) interactions were shown by using projector-augmented-wave methods^42^. The value of the convergence criteria for energy was set with 1.0 × 10^−5^ eV per cell and the force was relaxed below 0.02 eV Å^−1^ to optimize the ground-state atomic geometries. The kinetic cut-off energy of the plane-wave basis function was set as 400 eV. The total energy, electronic structures and stress/force relaxations were calculated by the Gaussian method. DFT-D3 method of Grimme with zero-damping function was used to characterize the van der Waals interactions between molecules and perovskite surface^43^. During geometry optimization, the bottom layer was fixed while the other atoms were fully relaxed. The adsorption energy (*E*_ads_) was calculated by the following equation:

$${E}_{{\rm{ads}}}={E}_{{\rm{total}}}-{E}_{{\rm{slab}}}-{E}_{{\rm{free}}\ {\rm{molecule}}}$$

where *E*_total_, *E*_slab_ and *E*_free molecule_ are the total energy of adsorption structures, the energy of clean slab models and the energy of the free molecules, respectively.

## Online content

Any methods, additional references, Nature Portfolio reporting summaries, source data, extended data, supplementary information, acknowledgements, peer review information; details of author contributions and competing interests; and statements of data and code availability are available at 10.1038/s41586-024-07705-5.

### Supplementary information

Supplementary Information This file contains Supplementary Figs. 1–46, Notes 1–12, Table 1 and References.

Supplementary Video 1 Adhesion strength of the bonded crystals after curing for 1 hour. We conducted adhesion test to assess the binding force of HUBLA between the two crystals after curing for 1 hour. The result shows that the adhesion strength of the bonded crystals can be estimated to be around 2 g.

Supplementary Video 2 Adhesion strength of the bonded crystals after curing for 2 hours. The result shows that when the curing time of two crystals increases to 2 hours, the adhesion strength of the bonded crystals can be estimated to be around 5 g.

Supplementary Video 3 Adhesion strength of the bonded crystals after curing for 8 hours. The result shows that when the curing time of two crystals increases to 8 hours, the adhesion strength of the bonded crystals can be estimated to be around 10 g.

Supplementary Video 4 Adhesion strength of the bonded crystals after curing for 24 hours. The result shows that when the curing time of two crystals increases to 24 hours, the adhesion strength of the bonded crystals can be estimated to be around 20 g.

Supplementary Video 5 Adhesion experiment using HUBLA dissolved in ethanol. HUBLA is dissolved in ethanol and used for bonding the perovskite crystals. The result indicates that ethanol can be used as a solvent for HUBLA.

## Data Availability

The data that support the findings of this study are available from the corresponding authors upon reasonable request.
