# Supplementary material for: Water- and heat-activated dynamic passivation for perovskite photovoltaics
Source: Nature. 2024 Jun 24;632(8024):294–300. doi: 10.1038/s41586-024-07705-5 (PMC11306093; doi:10.1038/s41586-024-07705-5)
Supplement: Supplementary file 1 — This file contains Supplementary Figs. 1–46, Notes 1–12, Table 1 and References. [file 41586_2024_7705_MOESM1_ESM.pdf]

nature portfolio

https://doi.org/10.1038/s41586-024-07705-5

Supplementary information

Water- and heat-activated dynamic passivation for perovskite photovoltaics

In the format provided by the authors and unedited

Supplementary Information for

Water- and heat-activated dynamic passivation for perovskite photovoltaics

Wei-Ting Wang[1,†], Philippe Holzhey[2,3,†], Ning Zhou[4,†], Qiang Zhang[5,†], Suer Zhou[2], Elisabeth A. Duijnstee[2], Kevin J. Rietwyk[3], Jeng-Yu Lin[6], Yijie Mu[1,7], Yanfeng Zhang[5], Udo Bach[3], Chun-Guey Wu[8], Hin-Lap Yip[4,9,10,11], Henry J. Snaith[2]*, Shien-Ping Feng[1,10]*

[1]Department of Systems Engineering, City University of Hong Kong, Kowloon, Hong Kong.

[2]Clarendon Laboratory, Department of Physics, University of Oxford, Oxford, United Kingdom.

[3]Australian Research Council Centre of Excellence in Exciton Science, Department of Chemical and Biological Engineering, Monash University, Clayton, Victoria 3800, Australia.

[4]Department of Materials Science and Engineering, City University of Hong Kong, Kowloon, Hong Kong.

[5]School of Chemistry, Xi'an Jiaotong University, Xi'an 710049, China.

[6]Department of Chemical and Materials Engineering, Tunghai University, Taichung City 407224, Taiwan.

[7]Department of Mechanical Engineering, The University of Hong Kong, Pokfulam Road, Hong Kong.

[8]Department of Chemistry and Research Center for New Generation Light Driven Photovoltaic Modules, National Central University, Taoyuan City 320317, Taiwan.

[9]School of Energy and Environment, City University of Hong Kong, Kowloon, Hong Kong.

[10]Hong Kong Institute for Clean Energy, City University of Hong Kong, Kowloon, Hong Kong.

[11]Center of Super-Diamond and Advanced Films, City University of Hong Kong, Kowloon, Hong Kong.

†These authors contributed equally to this work.

*Corresponding authors. Email: tony.feng@cityu.edu.hk (S.-P.F.); henry.snaith@physics.ox.ac.uk (H.J.S.)

Table of contents

Supplementary Fig. 1 6

Supplementary Fig. 2 7

Supplementary Note 1 8

Supplementary Fig. 3 9

Supplementary Fig. 4 10

Supplementary Note 2 11

Supplementary Fig. 5 12

Supplementary Fig. 6 13

Supplementary Note 3 14

Supplementary Fig. 7 16

Supplementary Fig. 8 17

Supplementary Table 1 18

Supplementary Fig. 9 19

Supplementary Fig. 10 20

Supplementary Note 4 21

Supplementary Fig. 11 22

Supplementary Fig. 12 24

Supplementary Fig. 13 25

Supplementary Fig. 14 26

Supplementary Note 5 27

Supplementary Fig. 15 29

Supplementary Note 6 30

Supplementary Fig. 16 31
Supplementary Fig. 17 32
Supplementary Note 7 33
Supplementary Fig. 18 34
Supplementary Fig. 19 35
Supplementary Fig. 20 36
Supplementary Fig. 21 37
Supplementary Fig. 22 38
Supplementary Fig. 23 39
Supplementary Note 8 40
Supplementary Fig. 24 41
Supplementary Note 9 42
Supplementary Fig. 25 43
Supplementary Fig. 26 44
Supplementary Fig. 27 45
Supplementary Fig. 28 46
Supplementary Fig. 29 47
Supplementary Fig. 30 48
Supplementary Fig. 31 49
Supplementary Fig. 32 50
Supplementary Fig. 33 51
Supplementary Fig. 34 52
Supplementary Note 10 53

Supplementary Fig. 35 54

Supplementary Fig. 36 55

Supplementary Fig. 37 56

Supplementary Fig. 38 57

Supplementary Fig. 39 58

Supplementary Fig. 40 59

Supplementary Note 11 60

Supplementary Fig. 41 61

Supplementary Fig. 42 62

Supplementary Note 12 63

Supplementary Fig. 43 64

Supplementary Fig. 44 65

Supplementary Fig. 45 66

Supplementary Fig. 46 67

Supplementary Video 1 68

Supplementary Video 2 69

Supplementary Video 3 70

Supplementary Video 4 71

Supplementary Video 5 72

References 73

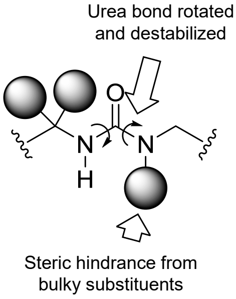

Supplementary Fig. 1 | Hindered urea bond (HUB). HUB is destabilized by bulky substituents, which induces bond rotation and decreases the conjugation effect.

The amide bond (-N(R)C(O)-) is generally stable because of its conjugation between the long electron pair on the nitrogen atom and the π-electrons on the carbonyl *p*-orbital. The steric hindrance by adding bulky groups can disturb orbital co-planarity and diminish the conjugation effect, which weakens carbonyl-amine interaction[1]. Hence, the HUB can dissociate into amine and isocyanate.

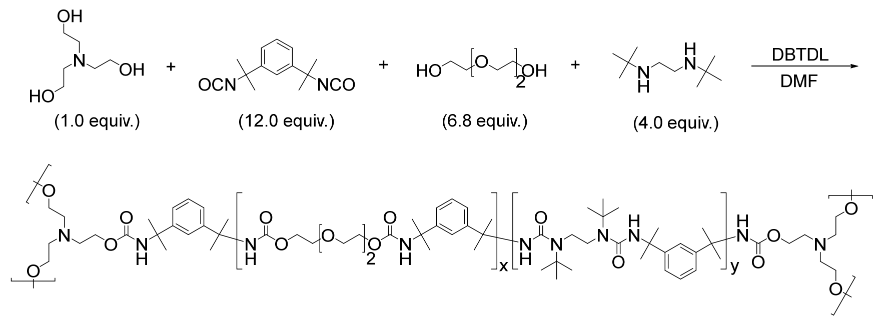

Supplementary Fig. 2 | Polymerization reaction of poly(urethane-urea) elastomer.

Supplementary Note 1

Preparation of poly(urea-urethane) and its self-healing behaviour

To show the self-healing ability of HUB (**Supplementary Fig. 3a**), a HUB-based poly(urea-urethane) elastomer was prepared; TEA, TMXDI, TEG, and *t*BEDA were mixed and cured at room temperature to form the elastomer[1]. Here, the fourteen elastomer cubes (5×5×5 mm^3; poly(urethane-urea)) were dyed with black color while another thirteen cubes were white, as shown in **Supplementary Fig. 3b**. As seen, after being placed at room temperature for 24 hours, the elastomer cubes could be reconnected through the cross-linked interfaces to build a Rubik's Cube. This self-healing property can be attributed to the dynamic reaction of HUBs with a dissociation-association reaction (the equilibrium reaction between HUB) at the interface[1]. Initially, the HUBs (**Supplementary Fig. 3c**) dissociated on the surfaces of the white and black cubes (**Supplementary Fig. 3d**), and the isocyanate and hindered amine groups at the interfaces would then react again to form HUBs (**Supplementary Fig. 3e**) when the two surfaces were attached.

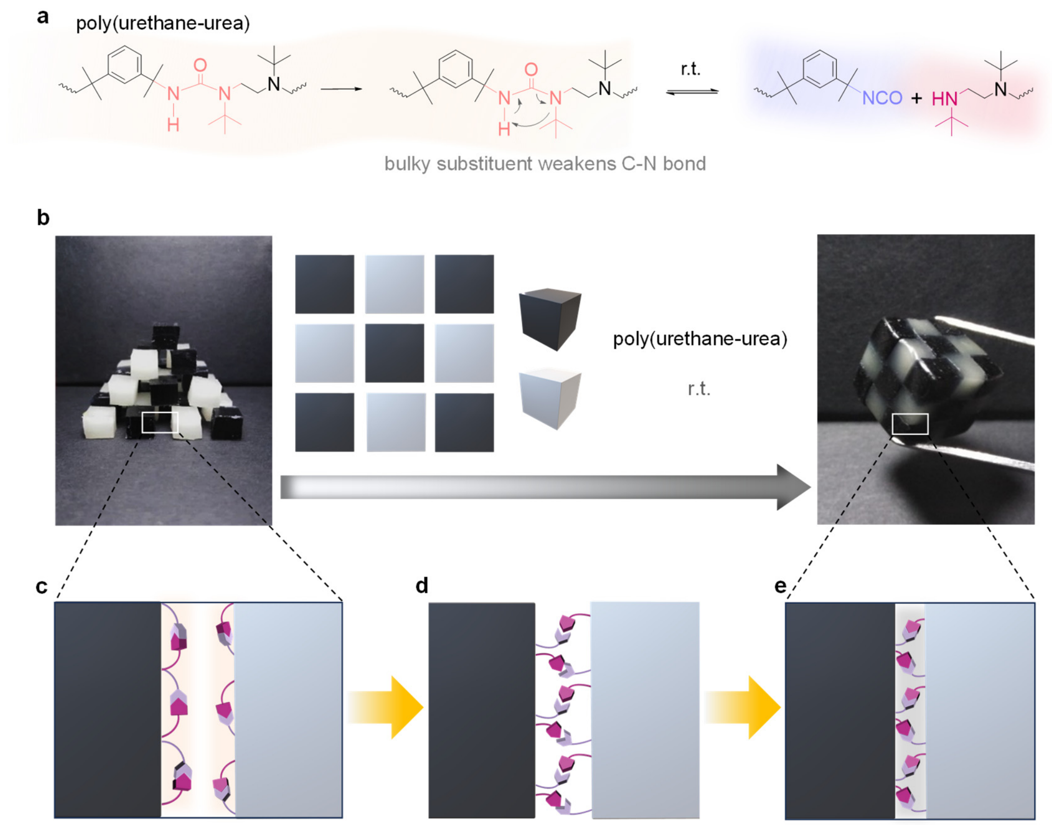

Supplementary Fig. 3 | Dynamic reaction of HUB and self-healing of Rubik's cube. (**a**) Chemical structures of HUB and its dynamic reaction. (**b**) Photographs of the self-healing experiment of HUB-based cross-linked poly(urethane-urea). (**c-e**) Illustration of the self-healing mechanism of HUB-based cross-linked poly(urethane-urea).

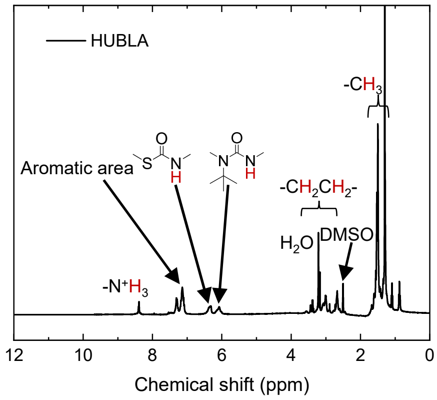

Supplementary Fig. 4 | Characterization of HUBLA. ^{1}H NMR (400MHz, DMSO-d_6): δ (ppm) = 8.39 (s, -N^+H_3), 7.31 and 7.14 (m, benzene ring), 6.33 (s, -SC(O)N(H)-), 6.07 (s, -N(C(CH_3)$_3$)C(O)N(H)-), 2.4-3.4 (m, -CH_2CH_2-), 1.50 and 1.30 (s, -CH_3). The ^{1}H NMR verifies the HUBLA structure. ATR-IR: 3285 and 1555 cm^{-1} (N-H); 2979 and 2868 cm^{-1} (C-H); 1649 cm^{-1} (C=O); 1487 cm^{-1} (C-N); 900 cm^{-1} (C-S). ESI-TOF MS: m/z = 851.41, calcd. 851.48 [M^+] for $C_{42}H_{72}ClN_8O_4S_2$.

Supplementary Note 2

Reaction between -NCO and weak Lewis bases

In general, IPA is used as a solvent to spin coat passivating materials on perovskite films, and the hydroxyl group (-OH) in IPA can react with the isocyanate group (-NCO) in NCO-AS to form urethane bonds (**Supplementary Fig. 5** illustrates the chemical formula for the reaction between NCO-AS and IPA). Conversely, when -NCO is protected with a weak Lewis base (such as *t*BEDA), it can be less prone to react with hydroxyl groups. To confirm this reaction, we conducted NMR spectroscopy analyses on solutions of IPA in combination with NCO-AS and HUBLA, respectively, to monitor their chemical structural changes (as shown in **Supplementary Fig. 5b** and **5c**). In particular, **Supplementary Fig. 5b** demonstrates that within 30 minutes of mixing NCO-AS with IPA, a signal corresponding to urethane bonds at around 7.13 ppm appeared, indicating that the isocyanate functional group of NCO-AS reacts with IPA[2]. In contrast, **Supplementary Fig. 5c** shows that within 2 hours of mixing HUBLA with IPA, we did not observe the formation of any new functional groups. And the characteristic peaks of HUBs and thiocarbamate bonds between 6.5 ppm to 6 ppm did not change, indicating that both the HUB and thiocarbamate bond can protect the NCO group from reacting with IPA.

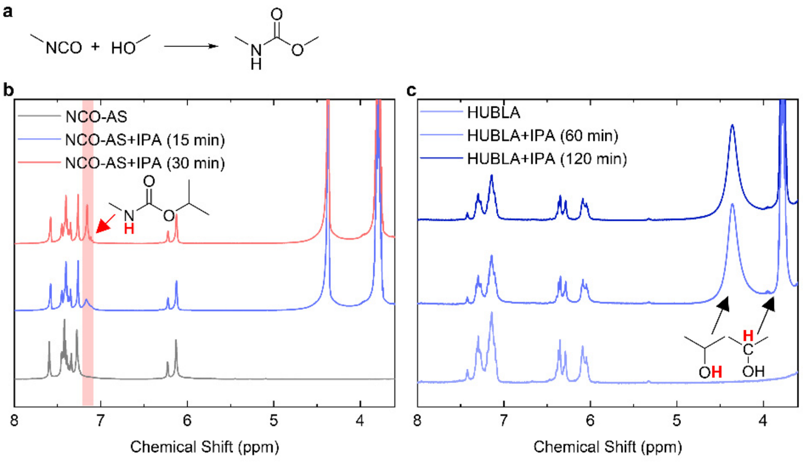

Supplementary Fig. 5 | Reaction between hydroxyl and isocyanate groups. (**a**) Chemical formula of reaction between hydroxyl and isocyanate groups. (**b**) ^{1}H NMR spectra of reaction between NCO-AS and IPA over a duration of 30 min. (**c**) ^{1}H NMR spectra of reaction between HUBLA and IPA over a duration of 120 min. For (**b**) and (**c**), IPA was added after 0 min.

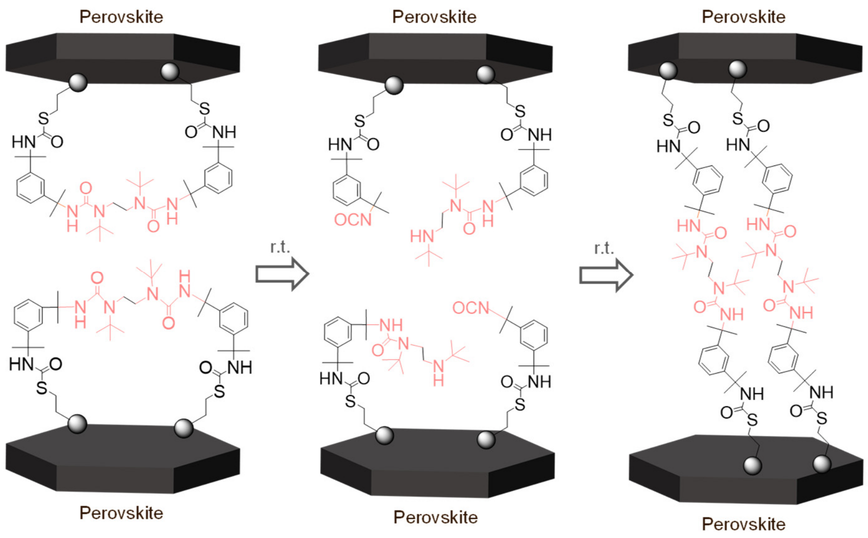

Supplementary Fig. 6 | Cross-linking mechanism of HUBLA. Two perovskite crystals generally cannot be bonded together because there is no interaction or bonding at the interface. In contrast, if the surfaces of perovskite crystals are coated with HUBLA, the HUB can dissociate and associate at the interface, and part of the HUBs can induce cross-linking at the interface between two crystals (the white ball represents $-N^{+}H_3$).

Supplementary Note 3

Crystal adhesion test

Two perovskite crystals can undergo dynamic exchange through HUBLA for adhesion. To test the possibility of pressure difference induced by solution filling, we initially conducted tests by using IPA solvent drops between the two crystals, as illustrated in **Supplementary Fig. 7**. The results indicate that, after bonding the two crystals for 8 hours, the IPA solution does not exhibit any adhesion strength between the two crystals. In addition, we conducted adhesion tests to assess the bonding force of HUBLA between the two crystals. We have recorded brief videos for reference, available in **Supplementary Videos 1** to **4**. The results demonstrate that when the bonding time for HUBLA between two crystals ranges from 1 hour to 24 hours, the adhesion strength of the bonded crystals can increase from 2 grams to 20 grams (**Supplementary Fig. 8**). Furthermore, we tested the feasibility of adhesion with other solutions, and we conducted experiments using twelve commonly used solvents. These experiments were carried out following the same procedure, and the summarized outcomes are presented in **Supplementary Table 1**. Additionally, we investigated the solubility of HUBLA in these solvents. The findings indicate that most of these solvents are unable to dissolve HUBLA and are unsuitable for bonding the two crystals. Among various solvents, HUBLA can be dissolved in DMF, ethanol, and IPA. The results demonstrate that when HUBLA is introduced into solvents with lower polarity, such as n-hexane and diethyl ether, it appears in a cloudy state (which can be referred to as a dispersed state). While HUBLA is introduced into water, it precipitates and adheres to the inner surface of the glass bottle. This is due to the structure of HUBLA, which contains both a bulky organic structure and ammonium salt groups, making it unsuitable for most of the

solvents for crystal adhesion experiments (all photographs of the dissolution experiments can be found in **Supplementary Fig. 9**). Furthermore, we also assessed the impact of DMF on the crystals, and the results showed that DMF rapidly degraded perovskite. Therefore, it cannot be used for HUBLA's crystal bonding experiments. The results indicate that IPA and ethanol are suitable solvents for HUBLA (**Supplementary Video 5** for the adhesion experiment using HUBLA dissolved in ethanol).

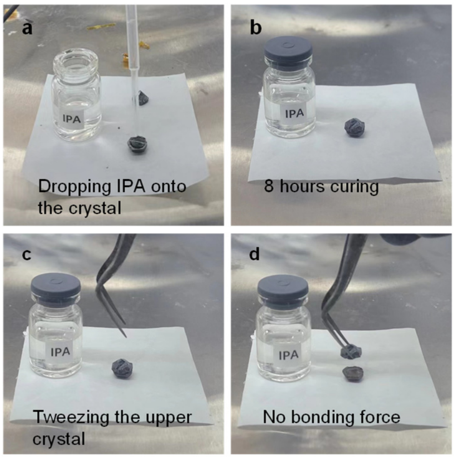

Supplementary Fig. 7 | Crystal adhesion experiment using IPA solvent. (**a**) Dropping IPA solvent on the crystal. (**b**) Placing another crystal on top and bonding for 8 hours. (**c**) Using a tweezer to lift the top crystal. (**d**) The two crystals separated without any adhesion.

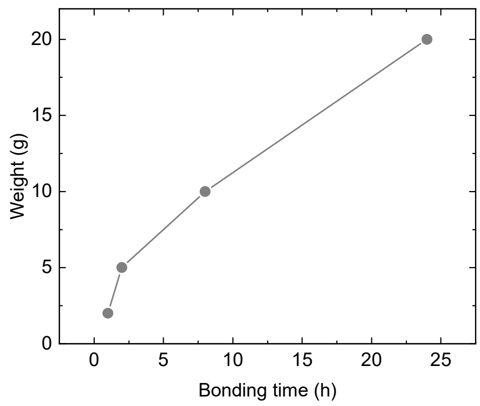

Supplementary Fig. 8 | The adhesive capacity of HUBLA between the two perovskite crystals.

Supplementary Table 1 | The summarized results of adhesion experiments.

Solvent	Bonding the two crystals (with HUBLA added in them)	Solubility of HUBLA
n-Hexane	No	Cloudy
Toluene	No	Cloudy
Benzene	No	Cloudy
Chloroform	No	Precipitate
Diethyl ether	No	Cloudy
Ethyl acetate	No	Cloudy
Tetrahydrofuran	No	Precipitate
Acetone	No	Precipitate
DMF	No (it would destroy the perovskite)	Soluble
Ethanol	Yes	Soluble
IPA	Yes	Soluble
H_2O	No (it would destroy the perovskite)	Precipitate

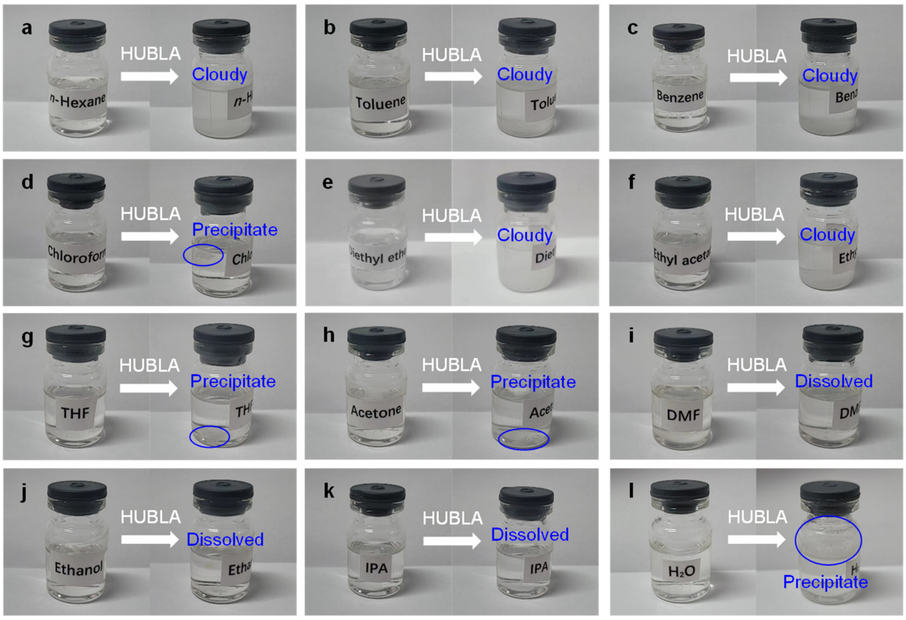

Supplementary Fig. 9 | HUBLA dissolution in different solvents. (**a**) Hexane. (**b**) Toluene. (**c**) Benzene. (**d**) Chloroform. (**e**) Diethyl ether. (**f**) Ethyl acetate. (**g**) Tetrahydrofuran. (**h**) Acetone. (**i**) DMF. (**j**) Ethanol. (**k**) IPA. (**l**) H_2O.

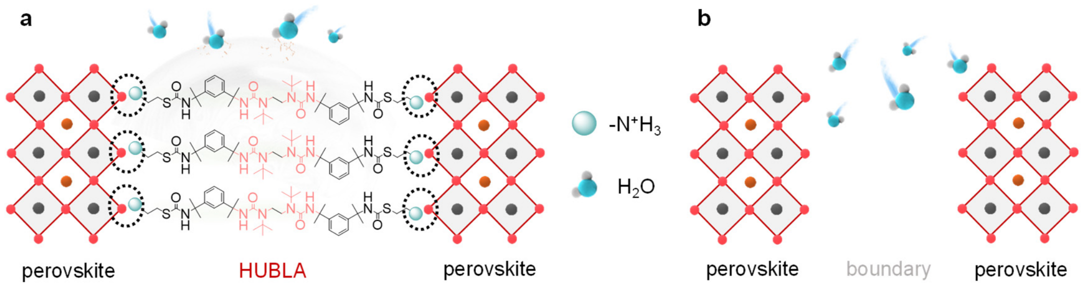

Supplementary Fig. 10 | Purposed behaviours of water molecules at perovskite grain boundaries. (**a**) Grain boundaries filled with HUBLA. (**b**) Pristine grain boundaries.

HUBLA can strongly interact with the perovskite, so HUBLA in the grain boundary of perovskite can act as a cross-linking agent to connect two perovskite grains. When HUBLA is in the grain boundary of perovskite, it can prevent the penetration of water.

Supplementary Note 4

Reaction between HUBLA and deuterium oxide (D_2O)

We added 15% (v%) D_2O into the HUBLA/DMSO-d_6 mixture (20 mg/mL) and measured the chemical changes of HUBLA at room temperature and 65 °C via ^{1}H NMR, respectively (**Fig. 1c**). The added D_2O can react with the water-sensitive -NCO group and further decompose into an amine[3], which prevents the association reaction, and thus the peak areas of HUB and thiocarbamate bond are decreasing. As shown in **Fig. 1c**, at room temperature, the peak area of HUB (blue curves) around 6.02 ppm dropped rapidly to 27.5% after 15 min and to 5.5% within one hour, which could be attributed to the hydrolysis of HUB. Subsequently, heating the solution to 65 °C, the peak area of the thiocarbamate bond (red curves) around 6.28 ppm dropped to 50.0% within 10 min and 19.2% within 40 min, which could be owing to the hydrolysis of thiocarbamate bond. Therefore, the highly reactive isocyanate group (-NCO) can be temporarily quenched by incorporating HUB and the thiocarbamate bond, while HUBLA can generate new passivators once environmental stressors appear.

a

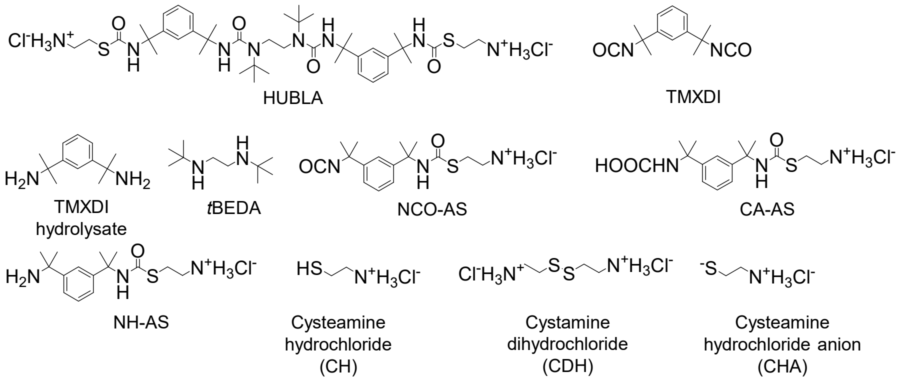

b Synthetic route of HUBLA:

$$2\text{TMXDI} + t\text{BEDA} \xrightarrow{+\,2\text{CH}} \text{HUBLA};$$

c Dynamic and hydrolysis reactions of HUBLA:

Step 1: HUBLA $\rightleftharpoons$ *t*BEDA + 2NCO-AS;

Step 2: $\text{NCO-AS} \xrightarrow{+\,H_2O} \text{CA-AS} \xrightarrow{-\,CO_2} \text{NH-AS};$

d Dynamic and redox reactions at elevated temperature in N_2 glovebox:

Step 1: HUBLA $\rightleftharpoons$ *t*BEDA + 2NCO-AS;

Step 2: $\text{NCO-AS} \overset{\Delta}{\rightleftharpoons} \text{TMXDI} + \text{CH};$

Step 3: $2\text{CH} \xrightarrow{+\,I_2} \text{CDH} + 2I^-;$

$\text{CDH} \xrightarrow{+\,Pb^0} 2\text{CHA} + Pb^{2+};$

$2\text{CHA} \xrightarrow{+\,I_2} \text{CDH} + 2I^-;$

} Redox shuttle

Supplementary Fig. 11 | Chemicals and reactions that are mentioned in this study. (**a**) All the chemical structures, including HUBLA, TMXDI, TMXDI hydrolysate, *t*BEDA, NCO-terminated ammonium salt (NCO-AS), carbamic acid group (-N(H)C(O)OH)-terminated ammonium salt (CA-AS), NH_2-terminated ammonium salt (NH-AS), cysteamine hydrochloride (CH), cystamine dihydrochloride (CDH), and cysteamine hydrochloride anion (CHA). (**b**) Synthetic route of HUBLA. (**c**) Dynamic and hydrolysis reactions of HUBLA at room temperature. In step 1, HUBLA reversibly dissociates into *t*BEDA and NCO-AS. In step 2, NCO-AS reacts with H_2O to produce CA-AS, which quickly decomposes to NH-AS and releases CO_2. (**d**) Dynamic and redox reactions at elevated temperature in N_2. In step 1, HUBLA reversibly dissociates into *t*BEDA and

NCO-AS. In step 2, NCO-AS reversibly dissociates into TMXDI and CH at elevated temperature. In step 3, CH reacts with I_2 to produce CDH and I^-. CDH reacts with Pb^0 to produce CHA and Pb^{2+}. CHA reacts with I_2 to produce CDH and I^-. CDH and CHA form a redox shuttle reaction.

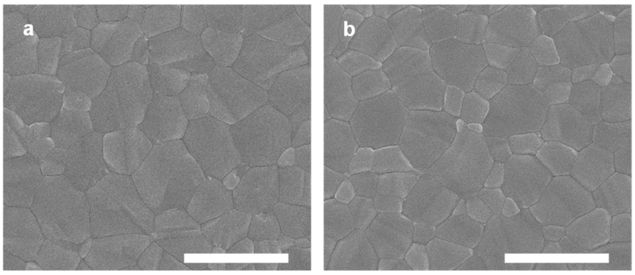

Supplementary Fig. 12 | Top-view SEM images of perovskite films. (**a**) Pristine perovskite. (**b**) Perovskite with HUBLA. The scale bar is 2 μm.

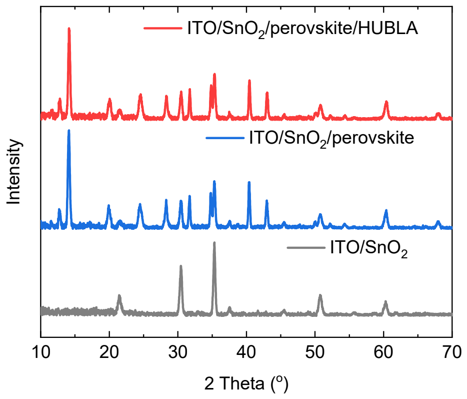

Supplementary Fig. 13 | XRD patterns of SnO_2, perovskite and perovskite/HUBLA films. The patterns showed similar peak intensities of perovskite and perovskite/HUBLA films.

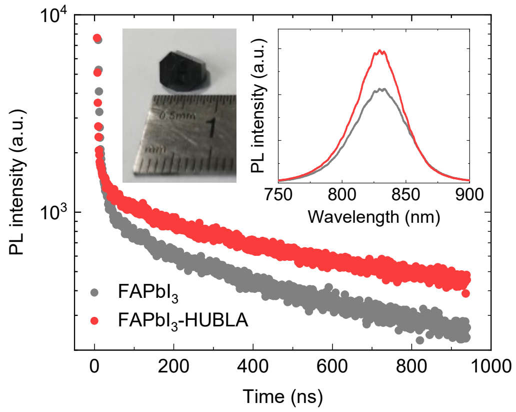

Supplementary Fig. 14 | Steady-state PL and TRPL of pristine and HUBLA-coated perovskite crystals. Insert figures are the photograph of the perovskite crystal and the steady-state PL.

Supplementary Note 5

Analysis of pulsed-voltage space-charge-limited-current (SCLC) measurement

SCLC is a commonly used steady-state technique that measures the dark current density-voltage (J-V) characteristic of a single-carrier device to study both the defect states in the bandgap of semiconductors and the charge carrier mobility. Perovskites are distinct from most conventional semiconductors as they exhibit characteristics of both electronic and ionic conduction. The presence of mobile ionic species has the potential to shield the bulk material from externally applied voltages, and thereby affects the total electronic current injection, transport and extraction. To circumvent this, we performed pulsed-voltage SCLC measurements[4], in which we pseudolocalized mobile ions during the dark J-V sweep, by applying very short voltage pulses (20 ms) and measuring the current before ions had time to move significantly. This enables the measurement of the intrinsic charge-carrier properties in perovskite semiconductors.

The extracted J-V curves for a semiconductor with traps are typically subdivided into three distinct regions. First is the Ohmic regime (1) at low voltages, where the current rises linearly with the voltage. After region (1), the J-V curve deviates from being linear at the onset voltage (V_{ons}), after which the slope of the J-V curve, $r = \frac{d(log(J))}{d(log(V))}$, is larger than two due to the filling of the trap states (2). Lastly, when all traps are filled, the drift current density profile for a single-carrier device follows the Mott-Gurney law, where the current density is proportional to the voltage squared (3). These three distinct regions only occur under certain specific conditions, which are detailed in the reference[4].

Region (2), with a slope larger than two, occurs if the traps are negatively charged when filled with electrons and if the density of traps is larger than the density of ionic space

charge. If this is the case, a lower-bound trap density value (n_t) can be calculated via: $n_t = \frac{2V_{ons}\varepsilon_0\varepsilon_r}{eL^2}$, where ε_0 is the vacuum permittivity, L is the crystal thickness, and e is the electron charge. ε_r is the dielectric constant of the material and is equal to 49.4 for $FAPbI_3$. The derivation of this onset voltage can be found in the reference[4]. We calculated a lower-bound trap density of 5.06×10^{10} cm^{-3} for the HUBLA-treated $FAPbI_3$ single crystal and 3.90×10^{11} cm^{-3} for the pristine $FAPbI_3$ single crystal. The great reduction in lower-bound trap density for the HUBLA-treated crystal confirms the healing of traps through HUBLA.

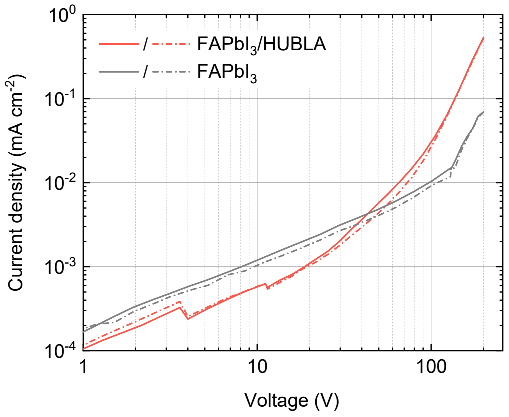

Supplementary Fig. 15 | Pulsed-voltage space-charge-limited-current measurements of perovskite crystals.

Supplementary Note 6

Identification of N 1*s* component peaks for XPS analysis

To clarify the binding energy of each component on perovskite film, we first assigned the N 1*s* XPS spectra of each monomer on the perovskite film, as shown in **Supplementary Fig. 16**, and used TMXDI hydrolysate to evaluate the N 1*s* component of the primary amine group ($-NH_2$). TMXDI and TMXDI hydrolysate (characterized by ^{1}H NMR in **Supplementary Fig. 17**) showed the N 1*s* components at ~400.0 and ~399.7 eV, assigned to the isocyanate group (-NCO) and the primary amine group ($-NH_2$), respectively. *t*BEDA showed the N 1*s* components at ~401.8 and ~399.4 eV, assigned to ammonia and secondary amine group (-NH-), respectively. Now, we consider the N 1*s* XPS spectra of perovskite/HUBLA aged at 25 °C and 65% RH. Note that the N 1*s* component of the isocyanate group (-NCO) maintained a low intensity, and thus, we did not fit it; in addition, the N 1*s* component of the primary amine group ($-NH_2$) was close to the amide bond (-N(R)C(O)-, 399.7 eV, **Fig. 2a**) in HUBLA and thus is assigned to amide group.

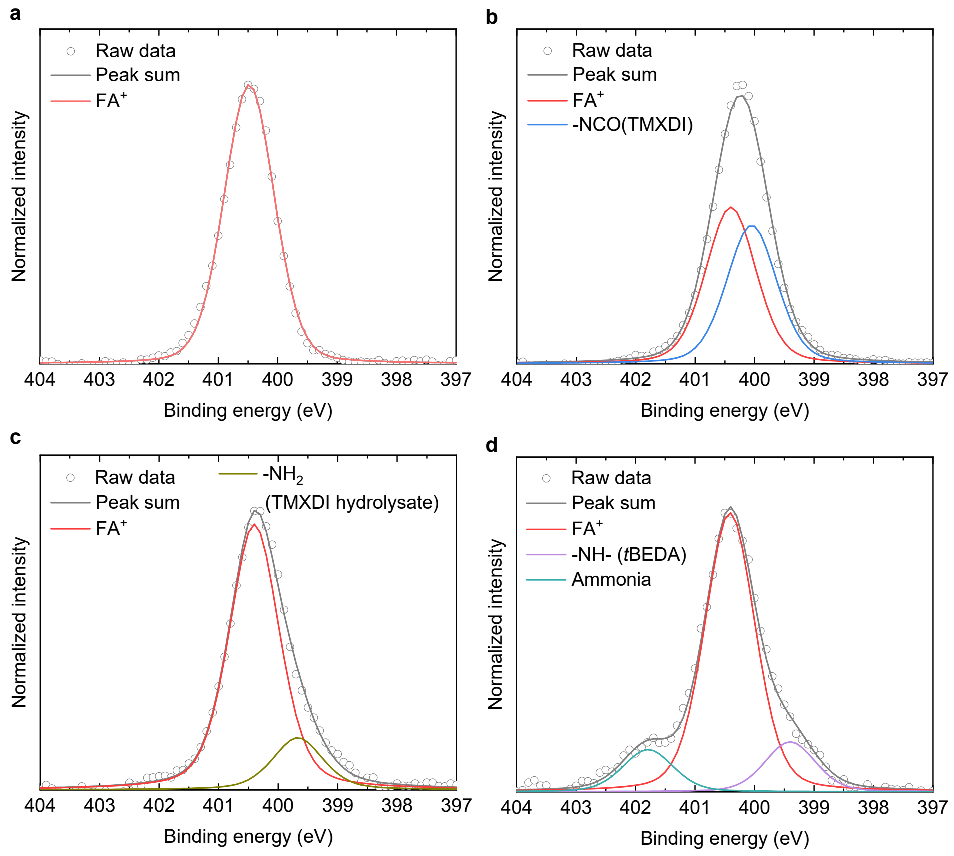

Supplementary Fig. 16 | N 1*s* XPS spectra of perovskite, perovskite/TMXDI, perovskite/TMXDI hydrolysate and perovskite/*t*BEDA films. (**a**) Perovskite film showing the N 1*s* component at ~400.5 eV. (**b**) Perovskite/TMXDI film showing two N 1*s* components at ~400.4 and ~400.0 eV that could be attributed to FA^+ and isocyanate (-NCO)[5], respectively. (**c**) Perovskite/TMXDI hydrolysate showing two N 1*s* components at ~400.4 and ~399.7 eV that could be attributed to FA^+ and primary amine (-NH_2)[6], respectively. (**d**) Perovskite/*t*BEDA films showing three N 1*s* components at ~401.8, ~400.4 and ~399.4 eV that could be attributed to ammonia, FA^+, and secondary amine (-NH-)[6], respectively.

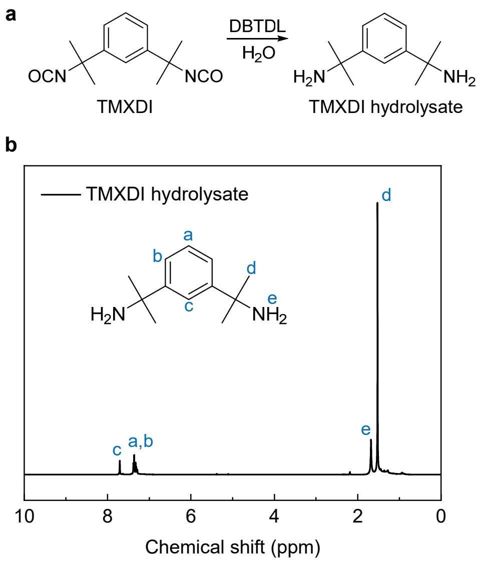

Supplementary Fig. 17 | Preparation and characterization of TMXDI hydrolysate. (**a**) Preparation of TMXDI hydrolysate. (**b**) ^{1}H NMR characterization of TMXDI hydrolysate.

Supplementary Note 7

Attenuated total reflection infrared (ATR-IR) spectroscopy of perovskite films wo/w HUBLA

The ATR-IR spectra of perovskite films w/wo HUBLA aged at 25 °C and 30% RH are shown in **Supplementary Fig. 18**. Compared to pristine perovskite film, the HUBLA-coated perovskite film showed a small peak at 1734 cm^{-1}, which is assigned to the carbamic acid group (-N(H)C(O)OH from CA-AS). Here, the characteristic peak from the functional group of CA-AS verifies the hydrolysis process of HUBLA by absorbing moisture on the surface of the perovskite[7-9]; note that the carbamic acid can automatically degrade into amine and CO_2. In addition, the characteristic peaks at 1350, 1469 and 1710 cm^{-1} could be assigned to the vibration of $-CH_3$ and $N-CH_3$, and the formamidinium antisymmetric stretching[10,11], respectively. The intensities of these peaks greatly decreased after 12 days (25 °C, 30% RH), indicating the decomposition of the $FAPbI_3$ crystal structure[12] with the possible formation of intermediate compounds[13]. By contrast, these characteristic peaks of the HUBLA-coated perovskite film still maintained most of their intensity after 12 days.

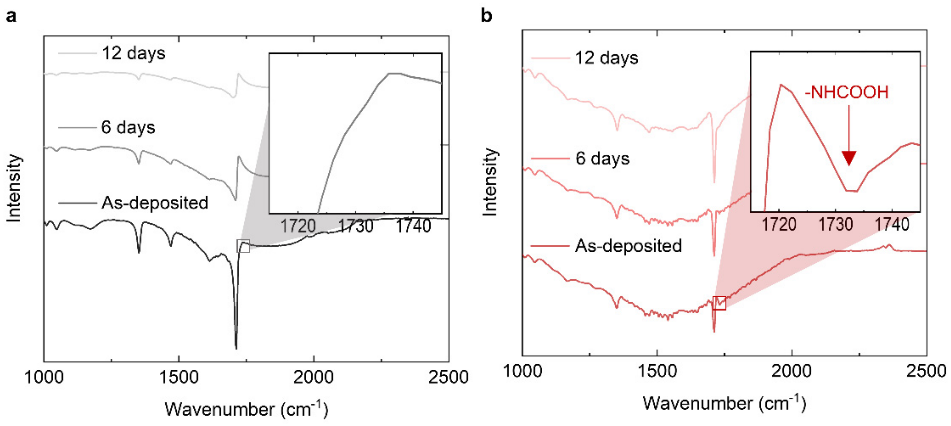

Supplementary Fig. 18 | ATR-IR spectra of the films. (**a**) Pristine perovskite film, and (**b**) perovskite/HUBLA film.

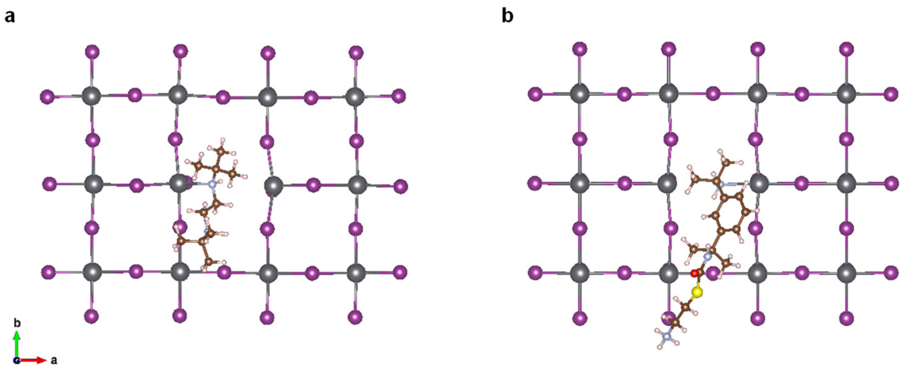

Supplementary Fig. 19 | Defects analysis and theoretical calculations. Theoretical models of perovskite with molecular surface passivation of (**a**) *t*BEDA, and (**b**) NH-AS. The nitrogen (N) atoms of *t*BEDA and NH-AS are found to interact with Pb atoms and passivate positively charged undercoordinated Pb^{2+} cations at interface[14].

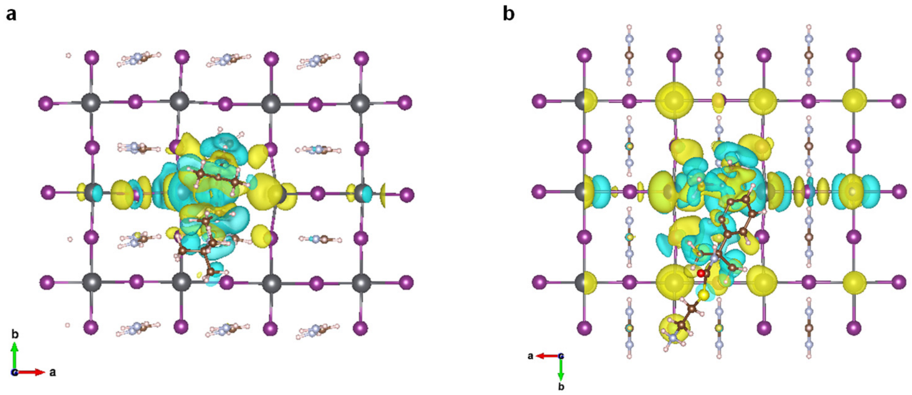

Supplementary Fig. 20 | The isosurface plots of the charge density difference. (**a**) *t*BEDA on perovskite, and (**b**) NH-AS on perovskite. Charge accumulation is in yellow and charge depletion is in blue.

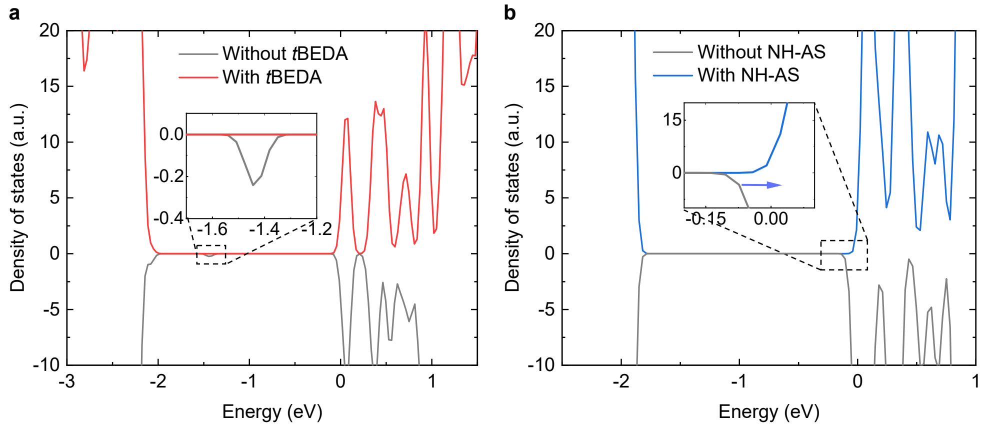

Supplementary Fig. 21 | Density of states of the perovskite films wo/w coating. (a) Pristine perovskite and perovskite/*t*BEDA, and (**b**) pristine perovskite and perovskite/NH-AS. The simulation finds that *t*BEDA and NH-AS can passivate the perovskite[15,16].

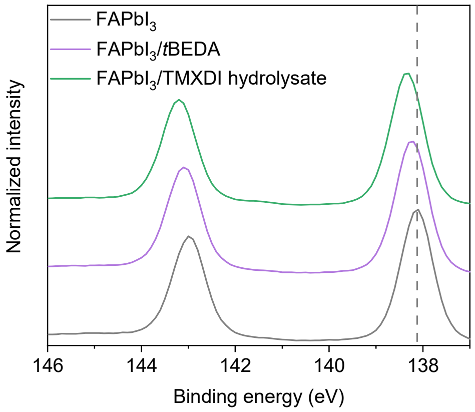

Supplementary Fig. 22 | Pb 4*f* XPS spectra of perovskite, perovskite/TMXDI hydrolysate, and perovskite/*t*BEDA films.

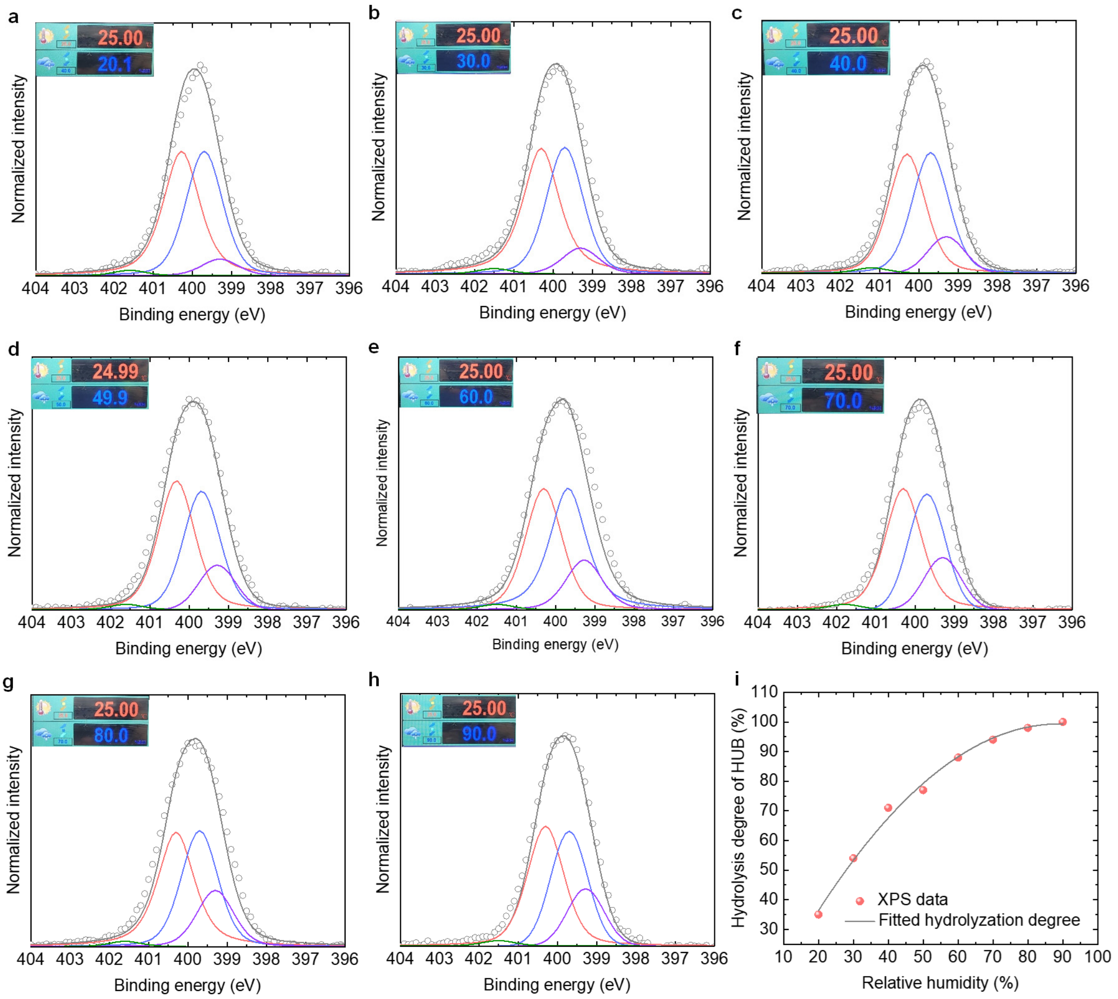

Supplementary Fig. 23 | The thresholds of water-activated characteristics. (**a-h**) N 1*s* regions of perovskite/HUBLA films aged at (**a**) 20%, (**b**) 30%, (**c**) 40%, (**d**) 50%, (**e**) 60%, (**f**) 70%, (**g**) 80%, and (**h**) 90% RH for 48 hours, respectively. (**i**) Summary of the conversion of HUB (%) under different humid conditions. The grey circles and curves represent the raw data and peak sum, respectively. The red, blue, green and purple curves represent N 1*s* components of FA^+, the amide bond (-N(R)C(O)-; also include the $-NH_2$), ammonia and -NH-, respectively.

Supplementary Note 8

Identification of S 2*p* component peaks for XPS analysis

To identify the binding energy of each component coated on perovskite film, we performed the S 2*p* XPS spectra of pristine HUBLA, perovskite/CH, and perovskite/CDH films, as shown in **Supplementary Fig. 24**. In all S 2*p* regions, each component exhibited a characteristic doublet with peaks separated about 1.2 eV apart and with an amplitude ratio of 2:1[17]. In particular, pristine HUBLA had the S 2*p* components at ~164.9 and ~163.7 eV (**Supplementary Fig. 24a**), corresponding to -SC(O)N(H)-. CH had the S 2*p* components at ~164.7 and ~163.5 eV (**Supplementary Fig. 24b**), corresponding to -SH. CDH had two S 2*p* components (**Supplementary Fig. 24c**); one was at ~164.9 and ~163.7 eV, assigned to -S-S-, and the other one was at ~161.8 and ~160.6 eV, which is $-S^{-}$[18]. Based on the binding energies of these monomers, we can analyze the S 2*p* XPS spectra of perovskite/HUBLA aged under 85 °C in N_2. Note that the S 2*p* components of -SH maintained a low intensity, and thus, we did not fit it; in addition, the S 2*p* components of -S-S- were close to that of -SC(O)N(H)- in HUBLA and thus are assigned to thiocarbamate bond.

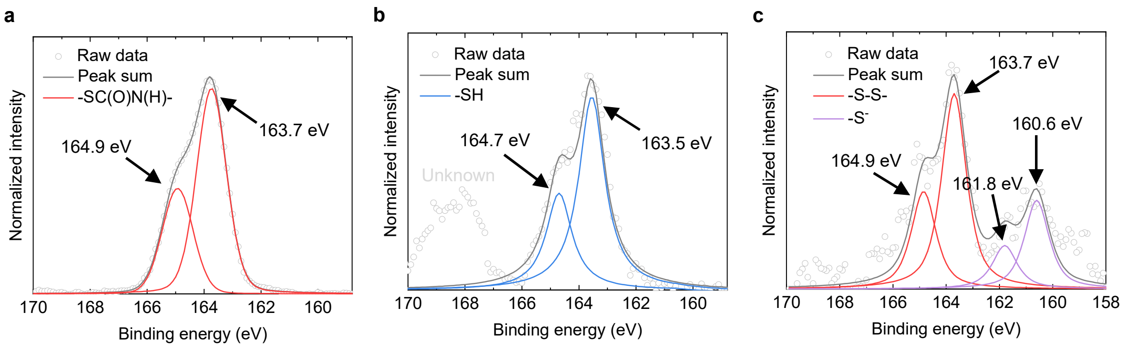

Supplementary Fig. 24 | S 2*p* XPS spectra of the perovskite films. (**a**) Pristine HUBLA, (**b**) perovskite/CH, and (**c**) perovskite/CDH films.

Supplementary Note 9

Redox reaction

HUBLA was added into 85 °C iodine solution, and the solution turned from dark brown to colourless (**Supplementary Fig. 25a** and **25b**) due to the oxidation of CH by I_2 and the production of CDH (^1H NMR spectra in **Supplementary Fig. 26**). Lead pellets were then added into the solutions (**Supplementary Fig. 25c**); the lead pellets were slowly dissolved only in the solution containing HUBLA (**Supplementary Fig. 25d**), suggesting that CDH can oxidize Pb^0 and generate Pb^{2+}.

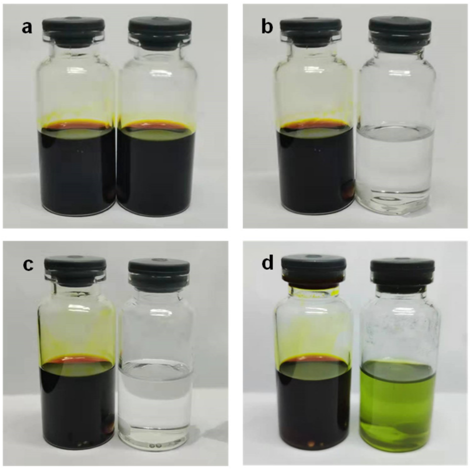

Supplementary Fig. 25 | Redox reaction of HUBLA. (**a)** Two sample bottles were filled with 10 mL DMF and 0.25 g I_2, and stirred at 85 ℃. (**b**) 1 g HUBLA was added into the right bottle and the solution became colorless. (**c**) Two lead pellets were added into both bottles. (**d**) The lead pellets in the right bottle were completely dissolved in the solution after stirring for 48 hours, and the lead pellets in the left bottle remained the same.

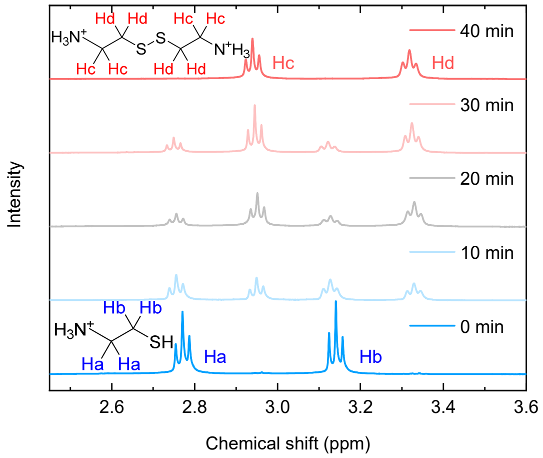

Supplementary Fig. 26 | ^{1}H NMR spectra of mixing CH and I_2 in D_2O at 85 °C. The thiol compound completely oxidized and formed the disulfide compound after 40 minutes.

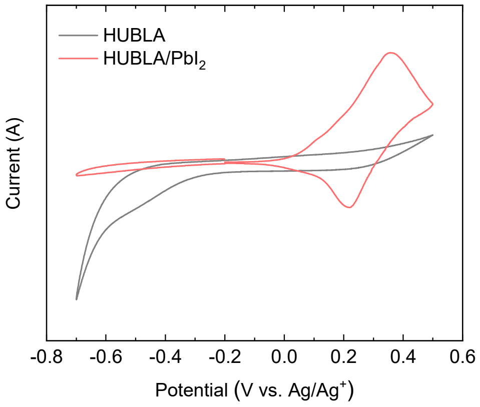

Supplementary Fig. 27 | CV curves of HUBLA and HUBLA/PbI_2 mixture.

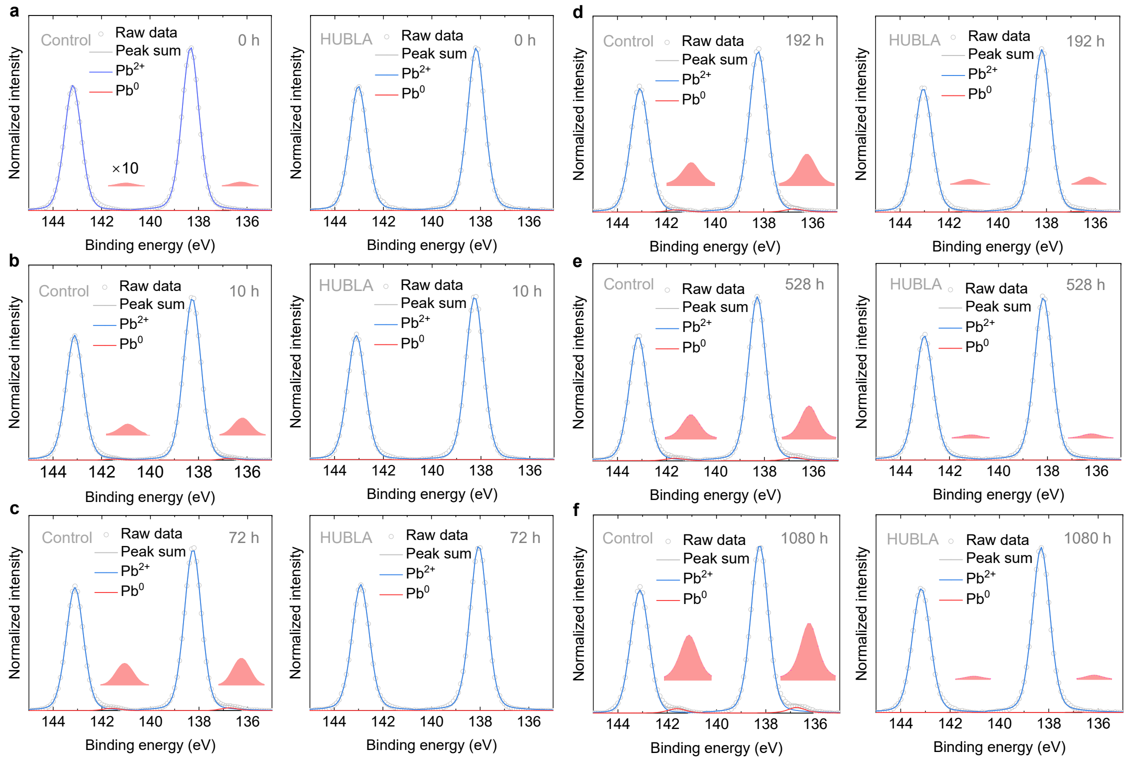

Supplementary Fig. 28 | Pb 4*f* XPS spectra during aging. Pb 4*f* XPS spectra of $FAPbI_3$ film and perovskite/HUBLA film aged at 85 °C in N_2 after (**a**) 0, (**b**) 10, (**c**) 72, (**d**) 192, (**e**) 528, and (**f**) 1080 hours, respectively. The intensity of Pb^0 components is enlarged in red peaks by the magnification of 10.

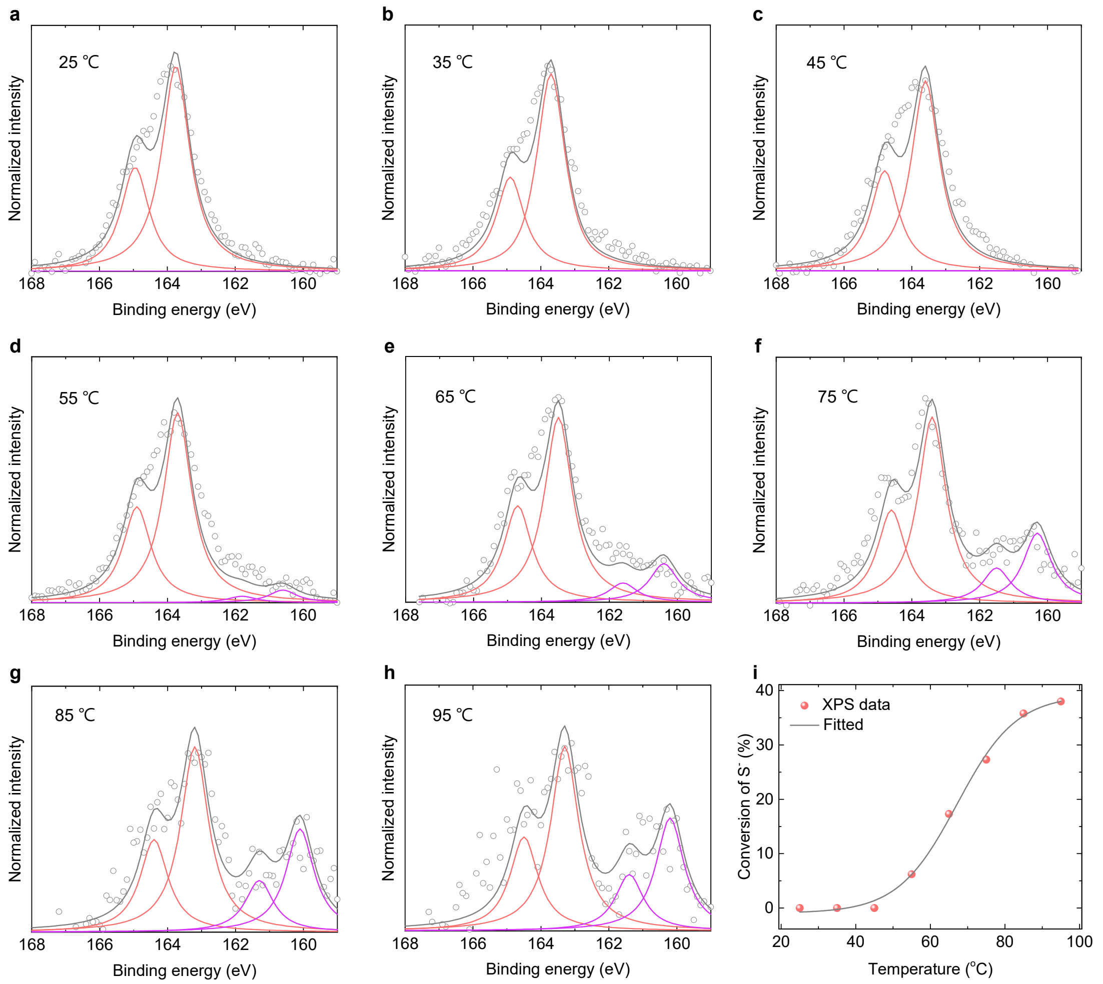

Supplementary Fig. 29 | The thresholds of heat-activated characteristics. (**a-h**) S 2*p* regions of perovskite/HUBLA films aged under (**a**) 25 °C, (**b**) 35 °C, (**c**) 45 °C, (**d**) 55 °C, (**e**) 65 °C, (**f**) 75 °C, (**g**) 85 °C, and (**h**) 95 °C for 1000 hours, respectively. (**i**) Summary of the conversion of -S$^-$ (%). The grey circles and curves represent the raw data and peak sum, respectively. The red and purple curves represent S 2*p* spectra of thiocarbamate bond (-SC(O)N(H)-; also include the -S-S-) and -S$^-$, respectively.

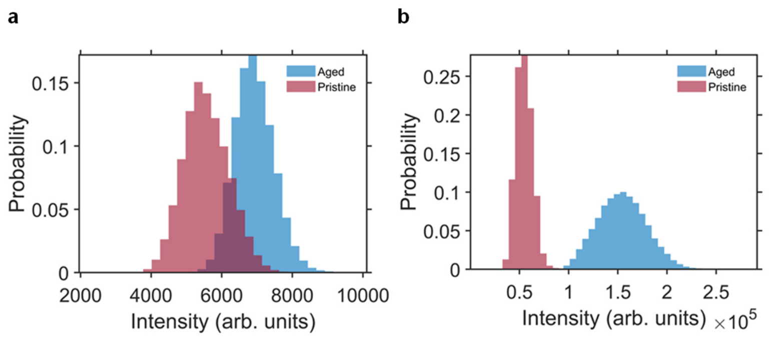

Supplementary Fig. 30 | Histograms for PL maps of perovskite films wo/w HUBLA. Histograms corresponding to **Fig. 2e** and **2f** show the distribution of the two areas, aged and pristine, for (**a**) control, and (**b**) HUBLA samples.

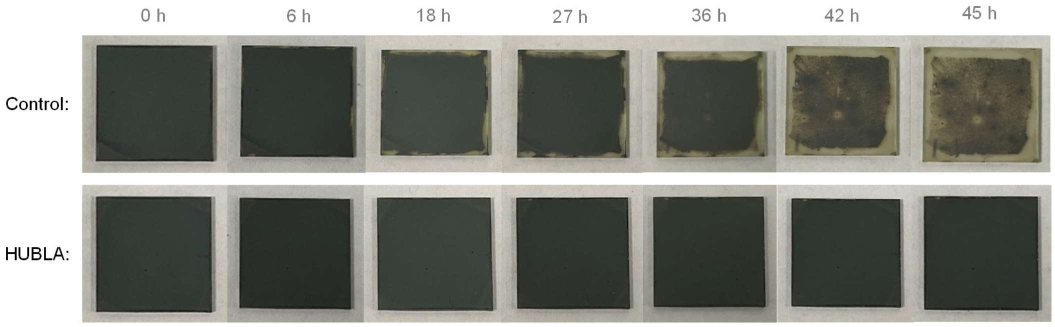

Supplementary Fig. 31 | Aging tests (25 °C, 60-70% RH) of perovskite films wo/w HUBLA. The upper and lower photographs are the control and HUBLA-coated perovskite films, respectively.

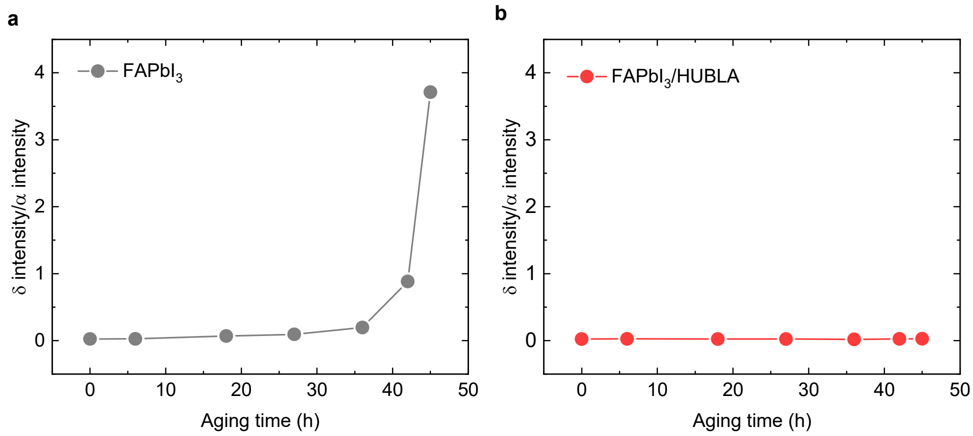

Supplementary Fig. 32 | δ phase/α phase ratios of the perovskite films. (**a**) Pristine perovskite, and (**b**) perovskite/HUBLA films.

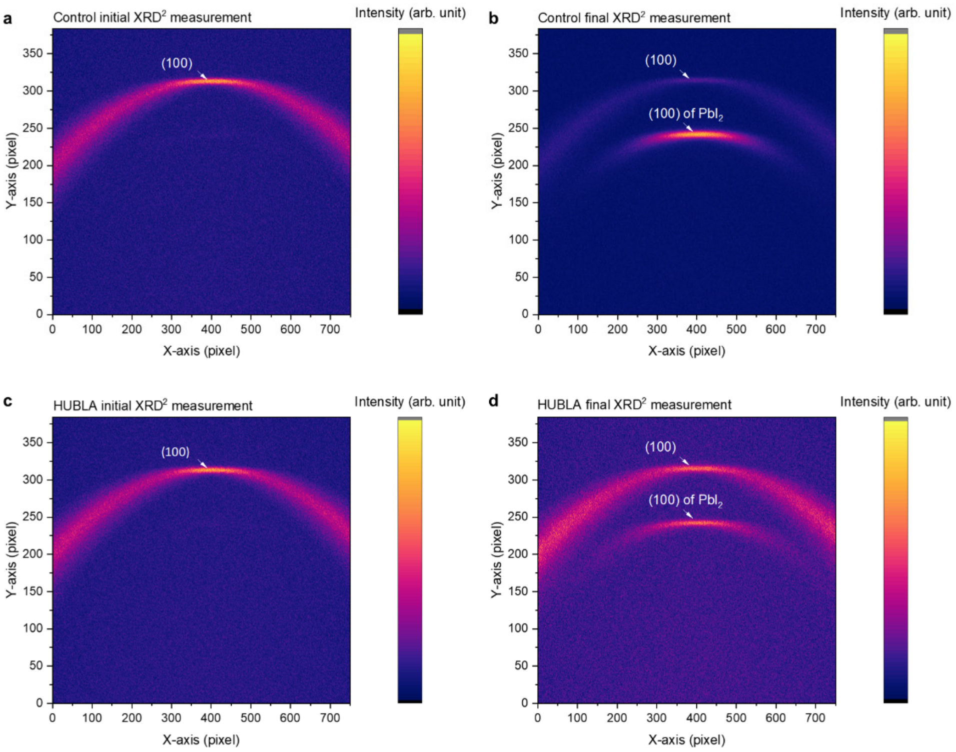

Supplementary Fig. 33 | XRD^2 measurements of the control and HUBLA films before and after 25.5 hours of aging at 85 °C in ambient air (~30% RH). (**a**, **b**) Control film and (**c**, **d**) HUBLA film. For **Fig. 3c** and **3d**, we measured roughly every minute a XRD^2 measurement and sum along the conic section of each peak to obtain a 1D XRD for each minute. **Supplementary Fig. 34** shows the change of the conic section for the PbI_2 peak and the change in the ratio of the (100) and PbI_2 peak.

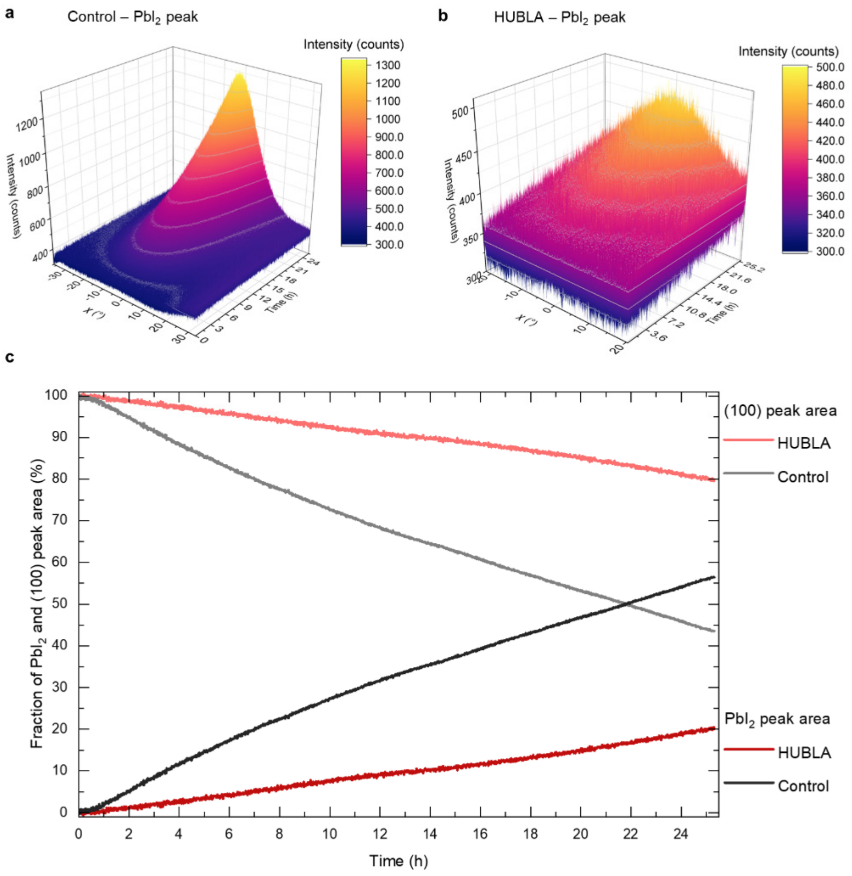

Supplementary Fig. 34 | The change along the azimuthal angle χ for the PbI_2 peaks. Change of the PbI_2 peak along the azimuthal angle χ for (**a**) the control, and (**b**) HUBLA films throughout the aging at 85 °C in ambient air (~30% RH). (**c**) The ratio of the integrated peak area of the (100) and PbI_2 peak after the background of each peak was subtracted. The measurements correspond to **Fig. 3c** and **3d** and **Supplementary Fig. 33**.

Supplementary Note 10

Aging test of $FA_{0.83}Cs_{0.17}Pb(I_{0.9}Br_{0.1})_3$ films

Here, we notice that the degradation of the perovskite thin films was likely accelerated by the presence of water and oxygen; therefore, it is not comparable to other published studies on the stability heated in N_2[19,20]. To decouple the influence of moisture from heat, we characterized another pair of thin films stored in ambient at 30% RH and room temperature. A series of optical microscopy images with illumination from below are shown in **Supplementary Fig. 35**; the corresponding ultraviolet-visible spectroscopy (UV-Vis) and XRD for each day of the same films are shown in **Supplementary Figs. 36** and **37**. The pristine thin film showed the first signs of degradation after 1 day stored at 30% RH, visible as bright spots in the optical microscopy images and a loss in absorption intensity around 450 to 525 nm. After 2 days, new peaks appeared around 9.5° and 12.7° in XRD. Furthermore, we could see a slight peak shift of the (100) diffraction and red-shift in absorbance onset for the pristine films, similar to Marchezi *et al*. who found the hexagonal phases form followed by the hydration of the perovskite[21]. That is likely correlated with the segregation of FA^+ and Cs^+ cations, as we also recently found via TOF-Sims 2D measurements[22]. In comparison, the perovskite thin film treated with HUBLA stayed the same until day 5, when the first bright spots appeared.

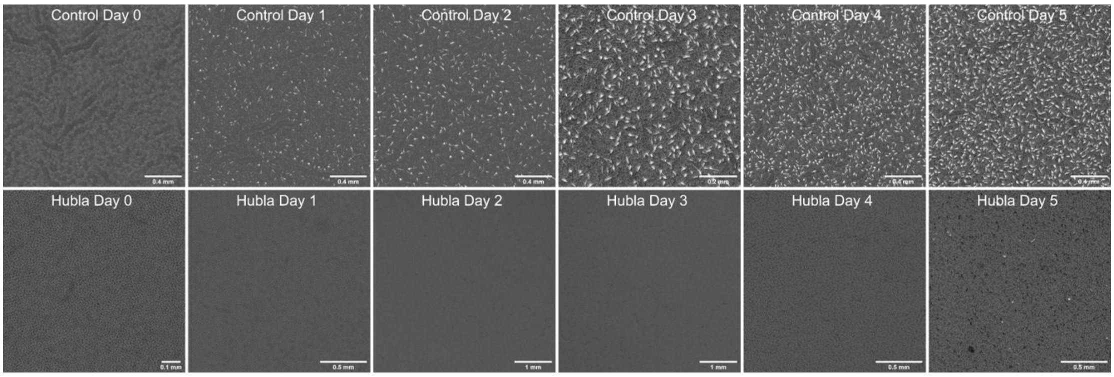

Supplementary Fig. 35 | Optical microscopy images for perovskite films. Perovskite thin films were stored at 30% RH. Corresponding UV-Vis and XRD measurements are shown in **Supplementary Fig. 36** and **37**.

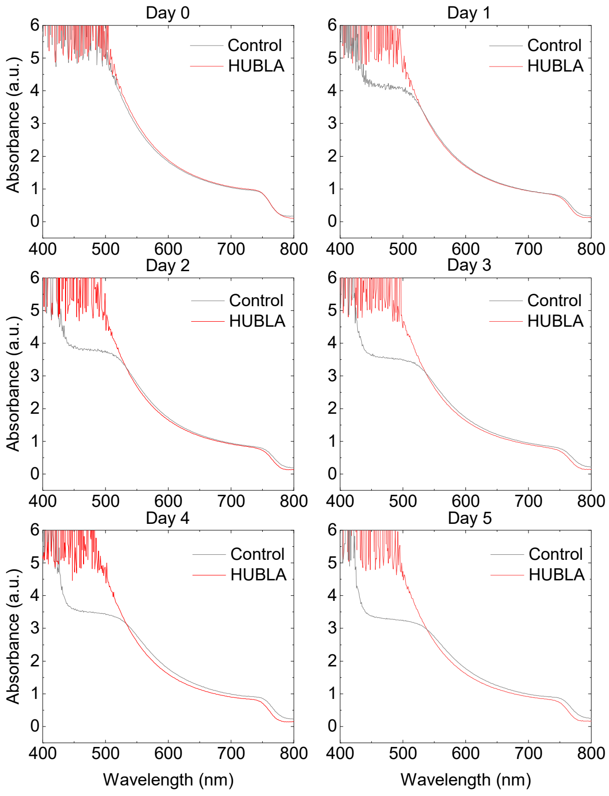

Supplementary Fig. 36 | Corresponding UV-Vis of the optical microscopy images. Perovskite films were stored at 30% RH. The loss in absorbance from 450 to 500 nm was likely a feature of the bright spots forming in **Supplementary Fig. 35** for the control films. The shift in absorbance onset can be interpreted as a sign of a smaller bandgap material forming like a FA^{+}-richer perovskite. That would also be consistent with the shift in (100) we observed in the XRD in **Fig. 3e and 3f**.

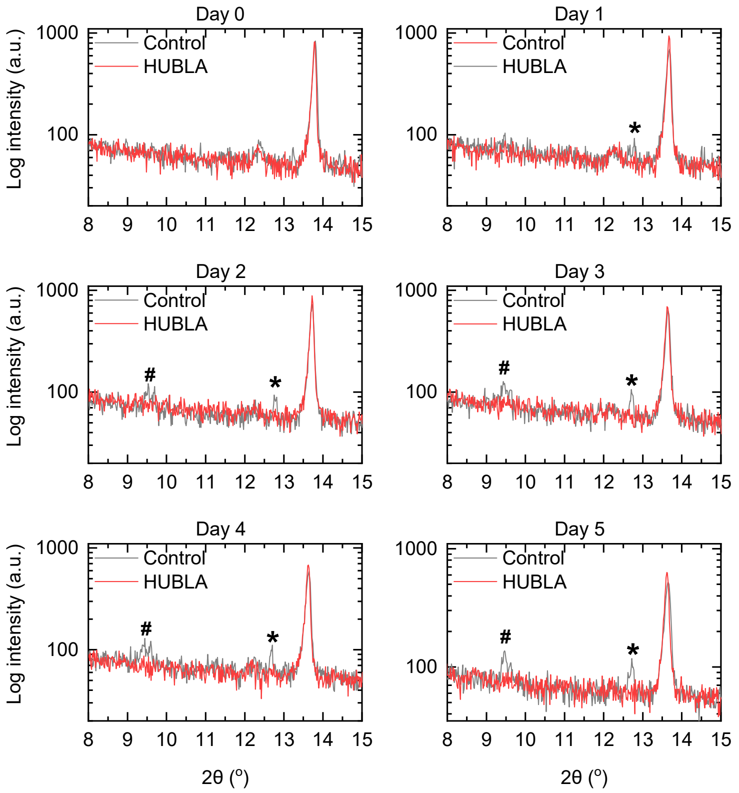

Supplementary Fig. 37 | Corresponding XRD of the optical microscopy images. Perovskite thin films were stored at 30% RH. The XRD peaks likely correspond to δ-$CsPbI_3$ (# symbol) and the lead halide (* symbol), which are marked in the XRD[21].

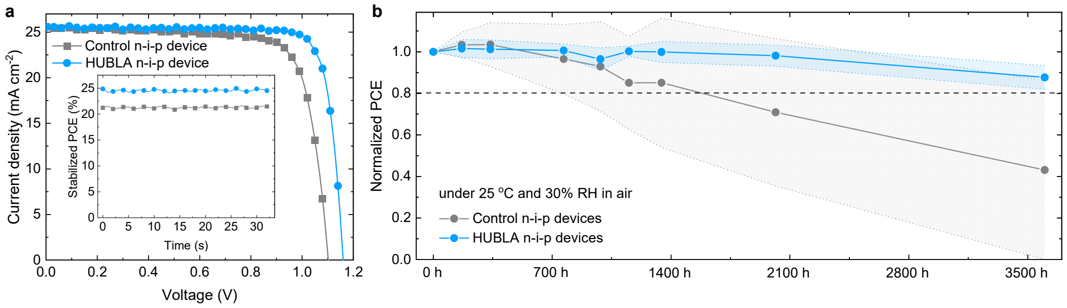

Supplementary Fig. 38 | Performance and stability of perovskite photovoltaics. (a) Best J-V curves with SPO. (**b**) Stability of unencapsulated n-i-p devices (Glass/ITO/SnO_2/perovskite/(wo/w) HUBLA/spiro-OMeTAD/MoO_3/Ag) in air (25 °C, 30% RH).

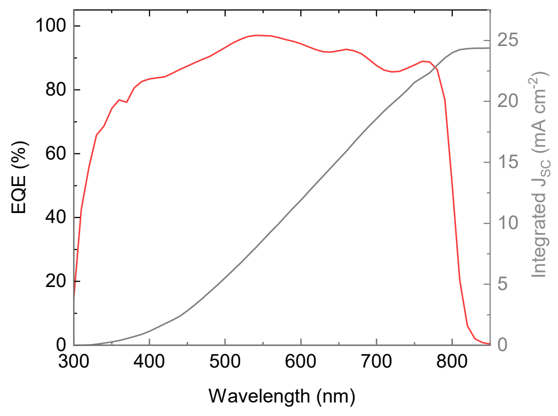

Supplementary Fig. 39 | EQE of HUBLA n-i-p device.

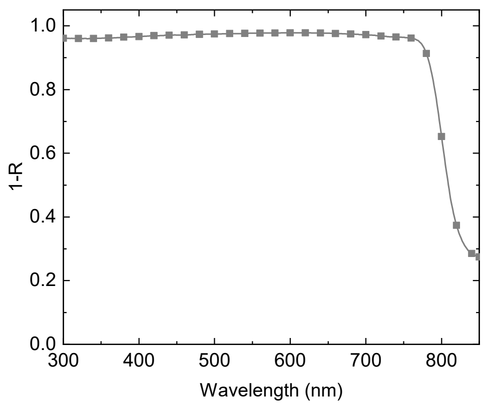

Supplementary Fig. 40 | Total absorbance (1-R) of Glass/ITO/perovskite.

Supplementary Note 11

N 1*s* XPS spectra of $FAPbI_3$/HUBLA film aged at 25 °C and 30% RH

To study the correlation between HUBLA hydrolysis and device performance, we performed the N 1*s* XPS spectra of $FAPbI_3$/HUBLA film aged at 25 °C and 30% RH during 480 hours, as shown in **Supplementary Fig. 41**. During 480 hours, the characteristic component of secondary amine (-NH- from *t*BEDA, purple curves) continuously increased. After 480 hours, the area of *t*BEDA was almost half of the HUBLA peak, indicating that HUB has almost dissociated and transformed into *t*BEDA and NH-AS. Therefore, HUBLA can continuously absorb water and release new passivation agents for at least 480 hours under 25 °C and 30% RH. The N 1*s* XPS spectra and the PCE result can be mutually verified under the same experimental conditions.

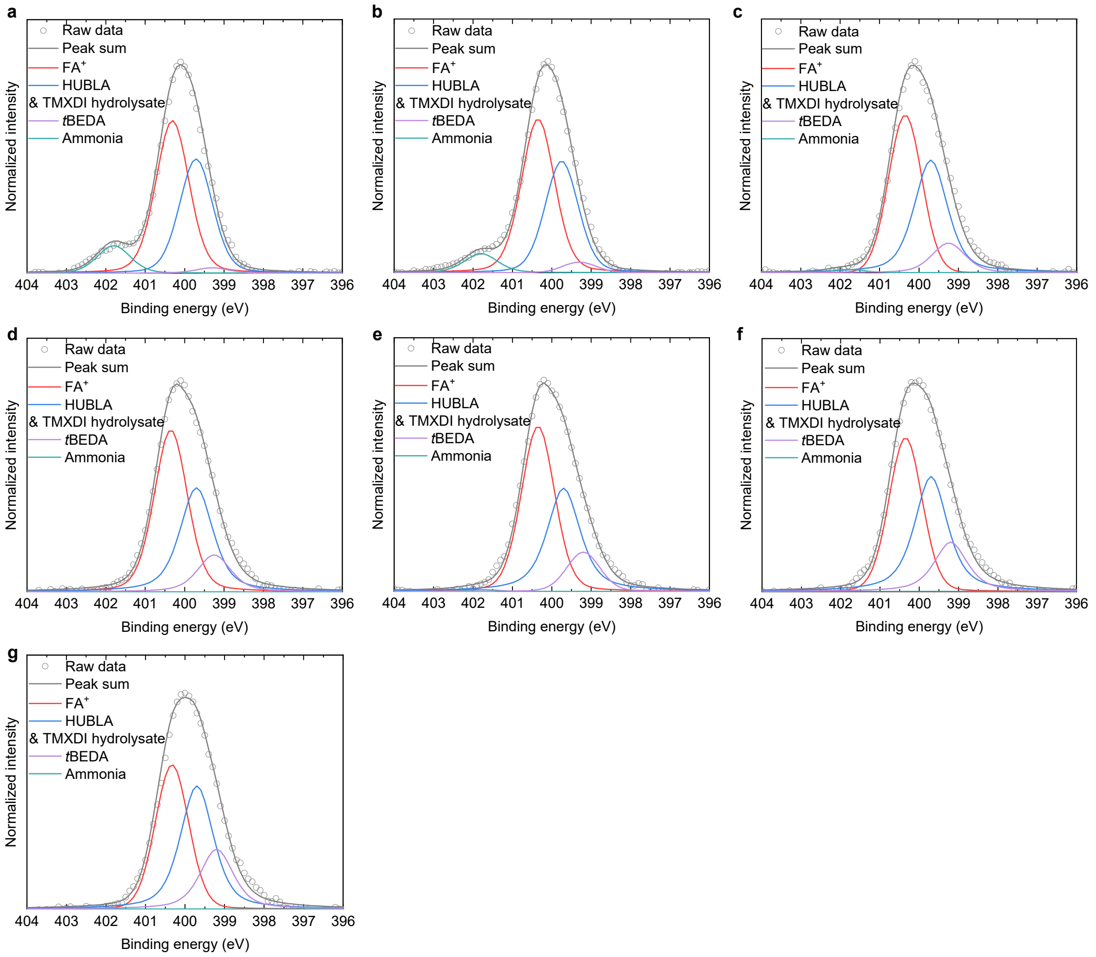

Supplementary Fig. 41 | N 1*s* XPS spectra of perovskite/HUBLA film in the aging test. The film aged at 25 °C under 30% RH after (**a**) 0, (**b**) 24, (**c**) 72, (**d**) 168, (**e**) 240, (**f**) 360, and (**g**) 480 hours.

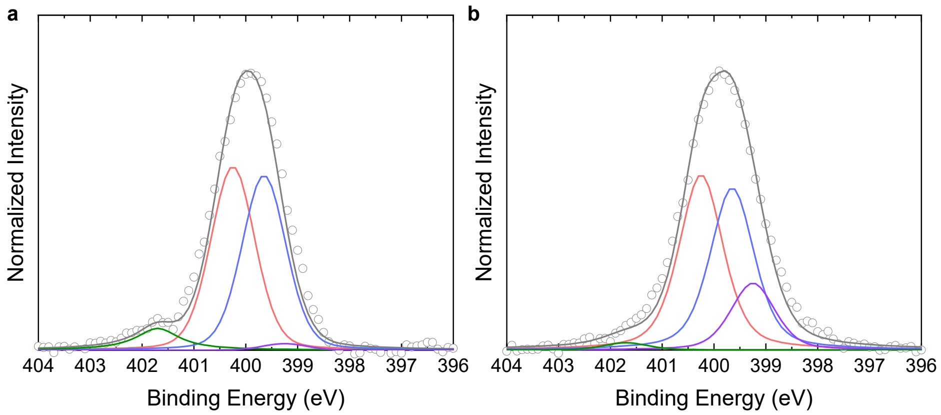

Supplementary Fig. 42 | N 1*s* XPS spectra of perovskite/HUBLA film aged at elevated temperature. The film was aged at 85 °C and 30% RH for (**a**) 0 hours, and (**b**) 120 hours. The grey circles and curves represent the raw data and peak sum, respectively. The red, blue, green and purple curves represent N 1*s* components of FA^+, the amide bond (-N(R)C(O)-; also include the $-NH_2$), ammonia and -NH-, respectively.

Supplementary Note 12

Thermal stability of p-i-n devices of around 2.5 years

We fabricated $FA_{0.83}Cs_{0.17}Pb(I_{0.9}Br_{0.1})_3$ composition p-i-n architecture with poly-TPD as a p-type layer and PCBM/BCP as an n-type layer[23,24] and Au as an electrode. Here, we measured the stability of the p-i-n devices at 85 °C in N_2 in the dark and chose to compare device performance to the most efficient HUBLA device after 1 day of aging as it induces the most significant increase in performance. As shown in **Supplementary Fig. 43**, the HUBLA device had a JV-scan of 17.5% (stabilized PCE of 16.8%) after 1 day, while it maintained a JV-scan of 16.1% (stabilized PCE of 14.4%, 86% of its stabilized PCE after 1 day) after 398 days (9552 hours) of aging at 85 °C in N_2.

Basically, all devices improved initially but then diverged during aging. The control devices reached their peak median performance at 240 hours (**Supplementary Fig. 44a**), and the HUBLA devices peaked at 768 hours (**Supplementary Fig. 44b**). We note that this initial improvement of p-i-n devices has already been studied in literature[23,25]. Given that the stability data is nonmonotonic, the consensus on stability data[26] suggests reporting the time till the devices reach 80% of their maximum PCEs (T_{S80}). The T_{S80} for the HUBLA devices was 11535.65 hours, and the retained initial fraction was 0.9048 after 21864 hours. In comparison, the twelve control devices had a T_{S80} of 2965 hours. The corresponding PV parameters for the HUBLA and the control devices are shown in **Supplementary Fig. 45**. The control devices showed a linear decay of the PV parameters after an initial increase; the initial increase in PCE of the HUBLA devices was more significant and over a longer timeframe. This different aging behaviour could arise from the redox shuttle reactions of HUBLA.

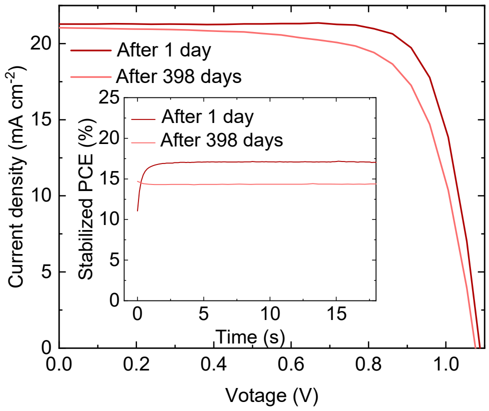

Supplementary Fig. 43 | Performance of p-i-n device aged after 1 and 398 days. J-V curves with stabilized PCE measurement for the most stable HUBLA p-i-n device (FTO/poly-TPD:F4-TCNQ/perovskite with $[BMP]^{+}[BF_4]^{-}$/HUBLA/PCBM/BCP/Au) after 1 and 398 days (9552 hours).

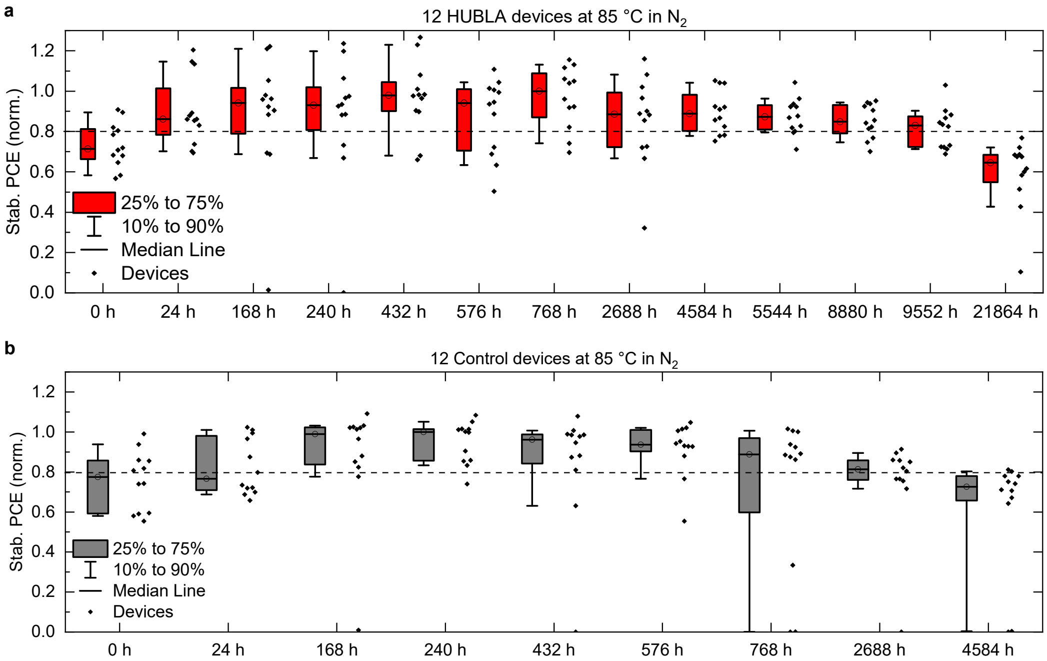

Supplementary Fig. 44 | Stability of 12 control and HUBLA devices aged at 85 °C in N_2. (**a**) Stability of twelve HUBLA p-i-n devices (same device structure as in **Supplementary Fig. 43**) aged at 85 °C in N_2 normalized to the peak median efficiency of 14.0% at 768 hours. They reached their T_{S80} at 11535.65 hours. (**b**) Stability of twelve control p-i-n devices. The devices were normalized to the peak efficiency at 240 hours of 16.0%. They reached their T_{S80} at 2965 hours.

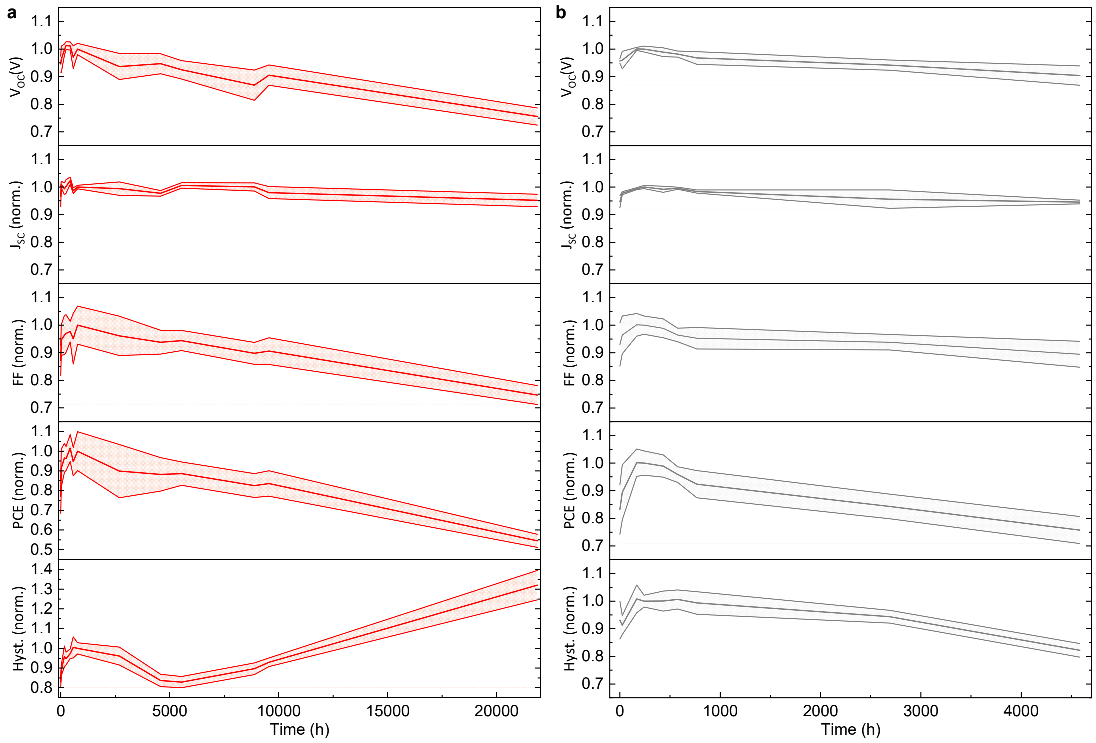

Supplementary Fig. 45 | Median PV parameters of the Supplementary Fig. 44. The upper and lower limits are the median absolute deviation (MAD). The PCE is from the JV-scan from short-circuit to open-circuit. FF, J_{SC} and V_{OC} are from the same JV-scan.

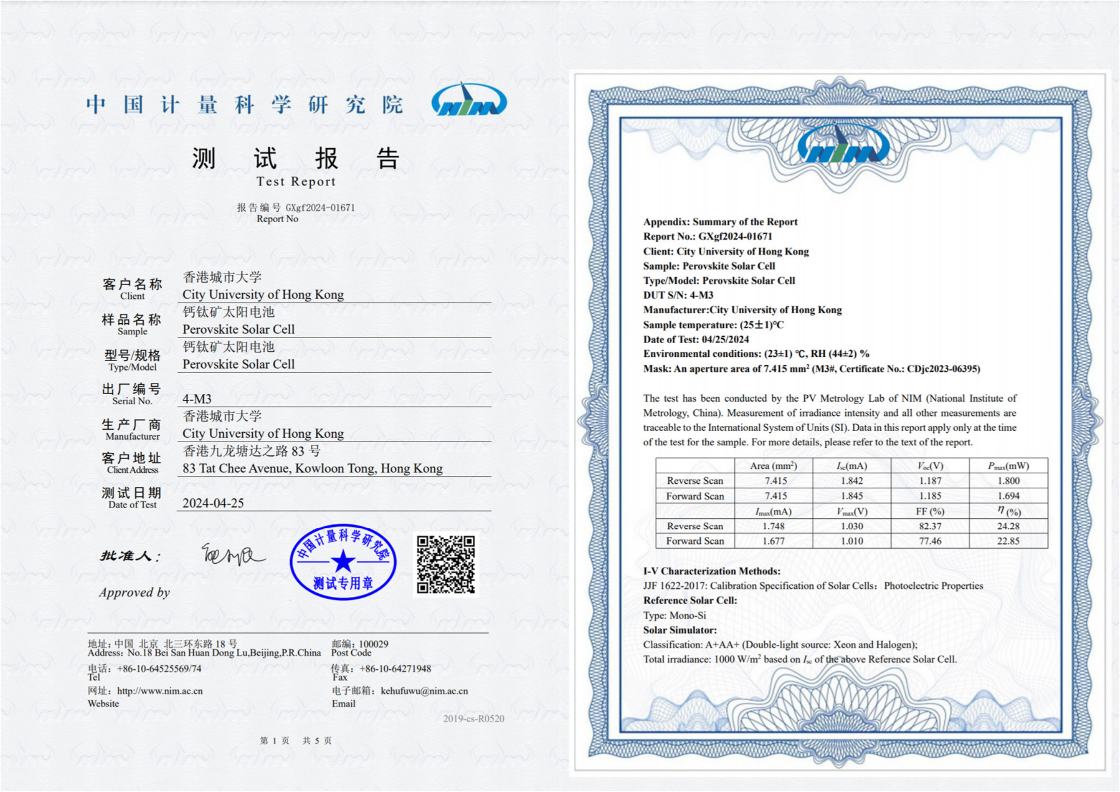

中 国 计 量 科 学 研 究 院

测 试 报 告

Test Report

报告编号 GXgf2024-01671
Report No

客户名称 Client: 香港城市大学 City University of Hong Kong

样品名称 Sample: 钙钛矿太阳电池 Perovskite Solar Cell

型号/规格 Type/Model: 钙钛矿太阳电池 Perovskite Solar Cell

出厂编号 Serial No.: 4-M3

生产厂商 Manufacturer: 香港城市大学 City University of Hong Kong

客户地址 Client Address: 香港九龙塘达之路 83 号 83 Tat Chee Avenue, Kowloon Tong, Hong Kong

测试日期 Date of Test: 2024-04-25

批准人: Approved by

中国计量科学研究院 测试专用章

地址：中国 北京 北三环东路 18 号
Address: No.18 Bei San Huan Dong Lu,Beijing,P.R.China

邮编：100029
Post Code

电话：+86-10-64525569/74
Tel

传真：+86-10-64271948
Fax

网址：http://www.nim.ac.cn
Website

电子邮箱：kehufuwu@nim.ac.cn
Email

2019-cs-R0520

第 1 页 共 5 页

Appendix: Summary of the Report
Report No.: GXgf2024-01671
Client: City University of Hong Kong
Sample: Perovskite Solar Cell
Type/Model: Perovskite Solar Cell
DUT S/N: 4-M3
Manufacturer:City University of Hong Kong
Sample temperature: (25±1)°C
Date of Test: 04/25/2024
Environmental conditions: (23±1) ℃, RH (44±2) %
Mask: An aperture area of 7.415 mm² (M3#, Certificate No.: CDjc2023-06395)

The test has been conducted by the PV Metrology Lab of NIM (National Institute of Metrology, China). Measurement of irradiance intensity and all other measurements are traceable to the International System of Units (SI). Data in this report apply only at the time of the test for the sample. For more details, please refer to the text of the report.

	Area (mm^2)	I_{sc}(mA)	V_{oc}(V)	P_{max}(mW)
Reverse Scan	7.415	1.842	1.187	1.800
Forward Scan	7.415	1.845	1.185	1.694
	I_{max}(mA)	V_{max}(V)	FF (%)	η (%)
Reverse Scan	1.748	1.030	82.37	24.28
Forward Scan	1.677	1.010	77.46	22.85

I-V Characterization Methods:
JJF 1622-2017: Calibration Specification of Solar Cells：Photoelectric Properties
Reference Solar Cell:
Type: Mono-Si
Solar Simulator:
Classification: A+AA+ (Double-light source: Xeon and Halogen);
Total irradiance: 1000 W/m² based on I_{sc} of the above Reference Solar Cell.

Supplementary Fig. 46 | Certified results of the inverted PSC with HUBLA.

Supplementary Video 1 | Adhesion strength of the bonded crystals after curing for 1 hour. We conducted adhesion test to assess the bonding force of HUBLA between the two crystals after curing for 1 hour. The result shows that the adhesion strength of the bonded crystals can be estimated to be around 2 g.

Supplementary Video 2 | Adhesion strength of the bonded crystals after curing for 2 hours. The result shows that when the curing time of two crystals increases to 2 hours, the adhesion strength of the bonded crystals can be estimated to be around 5 g.

Supplementary Video 3 | Adhesion strength of the bonded crystals after curing for 8 hours. The result shows that when the curing time of two crystals increases to 8 hours, the adhesion strength of the bonded crystals can be estimated to be around 10 g.

Supplementary Video 4 | Adhesion strength of the bonded crystals after curing for 24 hours. The result shows that when the curing time of two crystals increases to 24 hours, the adhesion strength of the bonded crystals can be estimated to be around 20 g.

Supplementary Video 5 | Adhesion experiment using HUBLA dissolved in ethanol. HUBLA is dissolved in ethanol and used for bonding the perovskite crystals. The result indicates that ethanol can be used as a solvent for HUBLA.

References

1. Ying, H., Zhang, Y. & Cheng, J. Dynamic urea bond for the design of reversible and self-healing polymers. *Nat. Commun*. **5**, 3218 (2014).
2. Madhi, A., Hadavand, B. S. & Amoozadeh, A. UV-curable urethane acrylate zirconium oxide nanocomposites: Synthesis, study on viscoelastic properties and thermal behavior. *J. Compos. Mater.* **52**, 2973–2982 (2018).
3. Ying, H. & Cheng, J. Hydrolyzable polyureas bearing hindered urea bonds. *J. Am. Chem. Soc.* **136**, 16974–16977 (2014).
4. Duijnstee, E. A. *et al.* Toward understanding space-charge limited current measurements on metal halide perovskites. *ACS Energy Lett.* **5**, 376–384 (2020).
5. Tardioa, S., Abel, M.-L., Carrb, R. H. & Watts, J. F. The interfacial interaction between isocyanate and stainless steel. *Int. J. Adhes. Adhes*. **88**, 1–10 (2019).
6. Oh, D. X., Shin, S., Lim, C. & Hwang, D. S. Dopamine-mediated sclerotization of regenerated chitin in ionic liquid. *Materials* **6**, 3826–3839 (2013).
7. Macedo, C. V. d. *et al.* Boron trifluoride catalyzed polymerisation of 2-substituted-2-oxazolines in supercritical carbon dioxide. *Green Chem*. **9**, 948–953 (2007).
8. Masuda, K., Ito, Y., Horiguchi, M. & Fujita, H. Studies on the solvent dependence of the carbamic acid formation from ω-(1-naphthyl)alkylamines and carbon dioxide. *Tetrahedron* **61**, 213–229 (2005).
9. Eftaiha, A. F. *et al.* Bis-tris propane in DMSO as a wet scrubbing agent: carbamic acid as a sequestered CO_2 species. *New J. Chem.* **41**, 11941–11947 (2017).
10. Liu, Z. *et al.* Enhanced crystallinity of triple-cation perovskite film via doping NH_4SCN. *Nanoscale Res. Lett.* **14**, 304 (2019).

11. Szostak, R. *et al.* Nanoscale mapping of chemical composition in organic-inorganic hybrid perovskite films. *Sci. Adv.* **5**, eaaw6619 (2019).
12. Zhou, Z., Pang, S., Ji, F., Zhang, B. & Cui, G. The fabrication of formamidinium lead iodide perovskite thin films via organic cation exchange. *Chem. Commun.* **52**, 3828–3831 (2016).
13. Wang, L. *et al.* A-site cation effect on growth thermodynamics and photoconductive properties in ultrapure lead iodine perovskite monocrystalline wires. *ACS Appl. Mater. Interfaces* **9**, 25985–25994 (2017).
14. Zhao, P., Kim, B. J. & Jung, H. S. Passivation in perovskite solar cells: a review. *Mater. Today Energy* **7**, 267–286 (2018).
15. Xia, J. *et al.* Deep surface passivation for efficient and hydrophobic perovskite solar cells. *J. Mater. Chem. A* **9**, 2919–2927 (2021).
16. Wang, T. *et al.* Deep defect passivation and shallow vacancy repair via an ionic silicone polymer toward highly stable inverted perovskite solar cells. *Energy Environ. Sci.* **15**, 4414–4424 (2022).
17. Kolesov, V. A. *et al.* Solution-based electrical doping of semiconducting polymer films over a limited depth. *Nat. Mater.* **16**, 474–480 (2017).
18. Chu, H. *et al.* Achieving three-dimensional lithium sulfide growth in lithium-sulfur batteries using high-donor-number anions. *Nat. Commun.* **10**, 188 (2019).
19. Turren-Cruz, S.-H., Hagfeldt, A. & Saliba, M. Methylammonium-free, high-performance and stable perovskite solar cells on a planar architecture. *Science* **362**, 449–453 (2018).
20. Schwenzer, J. A. *et al.* Thermal stability and cation composition of hybrid organic–

inorganic perovskites. *ACS Appl. Mater. Interfaces* **13**, 15292–15304 (2021).

21. Marchezi, P. E. *et al.* Degradation mechanisms in mixed-cation and mixed-halide $Cs_xFA_{1-x}Pb(Br_yI_{1-y})_3$ perovskite films under ambient conditions. *J. Mater. Chem. A* **8**, 9302–9312 (2020).
22. McMeekin, D. P. *et al.* Intermediate-phase engineering via dimethylammonium cation additive for stable perovskite solar cells. *Nat. Mater*. **22**, 73–83 (2023).
23. Bush, K. A. *et al.* 23.6%-efficient monolithic perovskite/silicon tandem solar cells with improved stability. *Nat. Energy* **2**, 17009 (2017).
24. Xu, J. *et al.* Triple-halide wide-band gap perovskites with suppressed phase segregation for efficient tandems. *Science* **367**, 1097–1104 (2020).
25. Saliba, M., Stolterfoht, M., Wolff, C. M., Neher, D. & Abate, A. Measuring aging stability of perovskite solar cells. *Joule* **2**, 1019–1024 (2018).
26. Khenkin, M. V. *et al.* Consensus statement for stability assessment and reporting for perovskite photovoltaics based on ISOS procedures. *Nat. Energy* **5**, 35–49 (2020).
